# The longitudinal bidirectional relationship between autistic traits and brain morphology from childhood to adolescence: a population-based cohort study

**DOI:** 10.1186/s13229-022-00504-7

**Published:** 2022-07-05

**Authors:** Melisa Durkut, Elisabet Blok, Anna Suleri, Tonya White

**Affiliations:** 1 Department of Child and Adolescent Psychiatry/Psychology, Erasmus MC–Sophia Children’s Hospital, University Medical Center Rotterdam, 3000 CB Rotterdam, The Netherlands; 2 The Generation R Study Group, Erasmus MC, University Medical Center Rotterdam, Rotterdam, The Netherlands; 3 Department of Radiology and Nuclear Medicine, Erasmus MC, University Medical Center Rotterdam, Rotterdam, The Netherlands

**Keywords:** Child/adolescent psychiatry, Neuroimaging, Development, Autism spectrum disorder, Neurodevelopmental disorders

## Abstract

**Objective:**

Autistic traits are associated with alterations in brain morphology. However, the anatomic location of these differences and their developmental trajectories are unclear. The primary objective of this longitudinal study was to explore the bidirectional relationship between autistic traits and brain morphology from childhood to adolescence.

**Method:**

Participants were drawn from a population-based cohort. Cross-sectional and longitudinal analyses included 1950 (mean age 13.5) and 304 participants (mean ages 6.2 and 13.5), respectively. Autistic traits were measured with the Social Responsiveness Scale. Global brain measures and surface-based measures of gyrification, cortical thickness and surface area were obtained from T_1_-weighted MRI scans. Cross-sectional associations were assessed using linear regression analyses. Cross-lagged panel models were used to determine the longitudinal bidirectional relationship between autistic traits and brain morphology.

**Results:**

Cross-sectionally, higher levels of autistic traits in adolescents are associated with lower gyrification in the pars opercularis, insula and superior temporal cortex; smaller surface area in the middle temporal and postcentral cortex; larger cortical thickness in the superior frontal cortex; and smaller cerebellum cortex volume. Longitudinally, both autistic traits and brain measures were quite stable, with neither brain measures predicting changes in autistic traits, nor vice-versa.

**Limitations:**

Autistic traits were assessed at only two time points, and thus we could not distinguish within- versus between-person effects. Furthermore, two different MRI scanners were used between baseline and follow-up for imaging data acquisition.

**Conclusions:**

Our findings point to early changes in brain morphology in children with autistic symptoms that remain quite stable over time. The observed relationship did not change substantially after excluding children with high levels of autistic traits, bolstering the evidence for the extension of the neurobiology of autistic traits to the general population.

**Supplementary Information:**

The online version contains supplementary material available at 10.1186/s13229-022-00504-7.

## Introduction

Autism spectrum disorder (ASD) has been associated with identifiable differences in structural brain morphology beginning early in life [1, 2]. There is increasing evidence that both autistic symptoms [3] and their related differences in brain morphology lie on a continuum in the general population [4, 5]. However, a limited number of studies have assessed developmental patterns of autistic traits and brain morphology in children. Extending this knowledge in large population-based studies and exploring potentially bidirectional developmental patterns have the potential to improve our understanding of the longitudinal interplay between neurobiology and autistic traits.

Previous studies assessing cortical morphology in children with ASD report mixed findings. Both gyrification and cortical thickness have been shown to have positive [6–9], negative [8, 10], or no associations [6, 11] in children with ASD. Some studies report no differences in surface area in individuals with ASD [6, 12], while others report smaller surface area [5, 7]. To date, only one study has evaluated the longitudinal relationship between gyrification and autistic traits. An abnormal increase in gyrification in frontal, temporal and parietal brain regions was observed in boys with ASD compared to controls from the age of 3–5 years [1]. Regarding total brain volume, studies have demonstrated overgrowth in young children with ASD [2, 8]. However, an atypical decrease in total brain volume [13] and widespread regions of accelerated cortical thinning have been observed from childhood into adulthood [8]. Overall, previous findings suggest that neurodevelopment remains atypical in ASD later in life.

While numerous studies have assessed the relationship between autistic symptoms and brain morphology, some key knowledge gaps remain. First, the few longitudinal studies available do not assess the interplay between brain and behavior from childhood to adolescence. The transition from childhood to adolescence is of specific interest when studying autistic symptoms and brain development, because this transition is characterized by enhanced social cognition coupled with structural brain changes [14]. Second, in our earlier work evaluating children with internalizing and externalizing symptoms, we found that it was actually behavioral symptoms that predicted brain morphology, rather than brain predicting behavior [15]. This suggests that persistent behaviors can potentially shape the brain. However, since autistic symptoms present early in life, the nature of the relationship with the brain might be different in comparison to internalizing and externalizing symptoms. No studies have assessed the bidirectional longitudinal relationship between autistic traits and brain morphology.

Within this backdrop, the primary aim of this study was to assess the longitudinal bidirectional relationship between autistic traits and brain morphology, from childhood to adolescence. Second, our goal is to study the association between cortical morphology and autistic traits and brain morphology in adolescents. Based on our earlier work [4, 5], we hypothesize that, during adolescence, higher levels of autistic traits are associated with less gyrification, smaller surface-area and larger cortical thickness in widespread regions in the brain.

## Methods

### Participants

The participants in this study were drawn from a large population based prospective cohort, the Generation R Study [16]. Magnetic resonance imaging (MRI) was performed in children at three different time points. The first wave of MRI data collection (referred to as W1) took place when the children were between 6 and 9 years of age (*N* = 1070), the second wave (W2) when the children were between 9 and 12 years of age (*N* = 3992), and the third wave (W3) when the children were between 13 and 16 years of age (*N* = 3625) [17, 18]. Children were excluded because of an incomplete T_1_-weighted scan, incidental findings on MRI that significantly altered brain morphology [19], dental braces, an unusable FreeSurfer reconstruction or missing data on autistic traits. For each sibling or twin pair, one sibling was randomly excluded. Children from W3 with useable MRI data and data on autistic traits composed the cross-sectional sample for this study (*N* = 1950). The longitudinal sample included children with both MRI data and measurements of autistic traits at W1 and W3 (*N* = 304). The flowchart in the supplement shows the inclusions and exclusions in detail (Additional file 1: Fig. S1).

### Assessment of autistic traits

To quantify autistic traits, parents completed an 18-item short form of the Social Responsiveness Scale (SRS) at both W1 and W3 [18]. The SRS is a parent-reported questionnaire assessing the social behavior of their child in the previous 6 months [18], with higher scores indicating more autistic traits [20]. The 18-item short form of the SRS has shown to be highly correlated with the full version of the SRS, ranging from 0.93 to 0.99 [21], and is a valid measure of both clinical and subclinical autistic traits [21].

In the Netherlands, a diagnosis of ASD is typically performed by a specialized multidisciplinary team from which the results are sent to the general practitioners (GP). The GPs hold the central medical records for medical diagnostic assessments and treatment. Thus, to identify children with a confirmed diagnosis of autism spectrum disorder (ASD), medical records were obtained from the children’s GPs. Medical records were obtained for all children who either: (i) scored above the clinical threshold score for the SRS; (ii) scored in the top 15% of the CBCL and above the clinical cutoff on the Social Communication Questionnaire. More details about this approach are described elsewhere [18] and in Additional file 1.

### Image acquisition

Prior to the actual MRI scanning procedure, all children participated in a mock scanning session to become acquainted with the MRI environment [17]. For W1, T_1_-weighted images were collected using a 3-Tesla General Electric Discovery MR750 system, (GE, Milwaukee, WI) [17]. For W3, a 3-Tesla GE Discovery MR750w (GE, Milwaukee, WI) system was used [18]. Both systems used an 8-channel receiving head coil. Information on the imaging parameters is presented in Additional file 1.

### Image processing and quality assurance

For both waves, MRI images were processed using FreeSurfer image analysis suite version 6.0 [22]. The processing stream has been described thoroughly elsewhere [22]. In brief, non-brain tissue was removed; voxel intensities were corrected for B_1_ inhomogeneities; anatomic regions were parcellated using the Desikan-Killiany atlas; and the cortex was reconstructed using a surface-based model [15]. Since artifacts, especially related to movement, can adversely influence the measures extracted from the T_1_-weighted images, all images were both visually inspected and run through an automated quality assessment algorithm [23, 24]. Images rated as either poor or unusable were excluded. Information on the image quality assurance is presented in Additional file 1.

### Covariates

The sex and date of birth for each child were derived from medical records obtained at birth. Age at time of completion of the SRS and the interval between the SRS and MRI scan at W1 and W2 were calculated based on the date of birth and the date of assessment. During pregnancy, data were collected by questionnaire on maternal smoking, maternal alcohol use and maternal education. Child national origin was based on the birth country of the parents. Handedness of the child was measured using the Edinburgh Handedness Inventory [23] at W1 and W3. Since a different MRI platform was used to acquire images at the two different waves (GE 750 Discovery for W1 and a GE 750w Discovery for W2), scanner could not be used as a covariate. Additional information about the covariates is provided in Additional file 1.

### Statistical analyses

All analyses were performed in R version 3.6.3 [25] and the R code can be accessed via the following link: https://github.com/tjhwhite/Gen_R_ASD_CLPM. Given the right-skewed distribution, SRS scores were square-root transformed (Additional file 1: Fig. S2) and thereafter SRS scores and brain measures were standardized to Z-scores (mean of zero and standard deviation of 1.0). Specific cortical and subcortical brain volumes included total brain, cortical gray matter, cerebral white matter, subcortical gray matter, cerebrospinal fluid (CSF), cerebellar cortex, cerebellar white matter, and the amygdala. Given that the current literature does not suggest lateralized effects of the amygdala, mean amygdala volume for the left and right hemisphere was calculated. In addition, mean values for gyrification, surface area and cortical thickness were extracted. Analyses were adjusted for confounders using a hierarchical approach. The first model was adjusted for sex, handedness, age at SRS and the SRS-MRI age difference. The second and main model was additionally adjusted for child national origin, maternal education, and maternal smoking and drinking during pregnancy. Missing data on covariates were imputed using the multiple imputation by chained equations (R package mice) with 30 imputed datasets and 30 iterations [26].

#### Cross-sectional analyses

We assessed the cross-sectional association between autistic traits and brain morphology measures collected at W3. The associations between autistic traits and global cortical and subcortical brain measures were calculated using linear regression analyses. Linear regression analyses testing the associations between autistic traits and vertex-wise measures of cortical thickness, surface area, and gyrification were performed using the Query Design Estimate Contrast R package (QDECR) [27]. Since ASD symptoms can be different between boys and girls [28], sex interactions were tested in the global and vertex-wise analyses by fitting an interaction term. When significant sex interactions were detected, models were stratified by sex. To account for multiple comparisons, Bonferroni corrections were applied for the eleven global brain measures (threshold *p* < 0.005) [29]. For the vertex-wise analyses, Gaussian Monte Carlo Simulations were used with the cluster-forming threshold set to *p* = 0.001. It has been shown that this threshold closely approaches a false positive rate of 0.05 [30]. Bonferroni corrections were used for analyzing both hemispheres with the cluster-wise threshold set to *p* < 0.025 [29].

#### Longitudinal analyses

The longitudinal, bidirectional relationship between autistic traits and brain morphology was investigated with cross-lagged panel models (CLPMs). The Lavaan package version 0.6-7 [31] was used to fit the CLPMs (Eq. 1).1

SRS_W1_ and SRS_W3_ are the scores of the SRS at W1 and W3, while MRI_W1_ and MRI_W3_ represent the brain metrics obtained at W1 and W3. Coefficient *ß*_CL-1_ represents the cross-lagged association between the SRS_W1_ and MRI_W3_, adjusted for the MRI_W1_. Conversely, coefficient *ß*_CL-2_ represents the cross-lagged association between MRI_W1_ and the SRS_W3_, adjusted for the SRS_W1_. Cross-sectional associations between the SRS score and brain metrics were also modeled (*ß*_CS-baseline_). Lastly, the autoregressive coefficients *ß*_AR-SRS_ and *ß*_AR-MRI_ were modeled, which represent the stability of the SRS score and brain metrics, respectively. In addition to the global measures, regions of interest (ROI’s) were included for the surface based measures, in which an association was found with the SRS in an independent sample of 1,954 children participating at W2, but not at W1 or W3 [5]. These ROIs included two clusters (lateral parietal and occipital) for gyrification, one cluster (superior frontal) for cortical thickness and two clusters (postcentral medial and orbitofrontal) for surface area. From these ROI’s the individual region mean for the corresponding metric (i.e., gyrification, cortical thickness or surface area) was calculated at W1 and W3 in the subgroup of children with longitudinal data. To control for false positives due to multiple comparisons, Bonferroni corrections were applied correcting for sixteen different models with a threshold set to *p* < 0.003 [29].

#### Sensitivity and non-response analyses

To investigate the robustness of the results, sensitivity analyses were performed. The first sensitivity analysis further adjusts model 2 for attention problems and child IQ, to determine whether the results are independent of IQ and attention problems. We adjusted for attention problems since these behaviors are highly comorbid in children with autistic symptoms [32] and to be consistent with our earlier work [4, 5]. Since we earlier found a relationship between neuropsychological performance and autistic symptoms [33], we also adjusted for IQ. In a second sensitivity analysis, children with the highest level of autistic traits and/or ASD were excluded to assess whether the relationship between autistic traits and brain morphology was similar in those with lower symptomatology. For this analysis, children with a confirmed ASD diagnosis and children with an SRS weighted total score above 1.078 for boys and 1.000 for girls, the cutoffs recommended for population-based screening [21], were excluded. In a third sensitivity analysis, we additionally corrected the vertex-wise analyses of gyrification, surface area and cortical thickness for mean gyrification, mean surface area and mean cortical thickness, respectively. In a fourth sensitivity analysis, we additionally corrected the longitudinal analyses of the global measures for total brain volume, and the ROI’s of gyrification, surface area and cortical thickness for mean gyrification, mean surface area and mean cortical thickness, respectively. The third and the fourth sensitivity analyses were performed to better distinguish regional from global effects. In a fifth sensitivity analysis, we performed a longitudinal whole-brain data driven approach, using all ROIs from the Desikan-Killiany atlas. To control for false positives due to multiple comparisons, we applied Bonferroni corrections correcting for 34 different models with a threshold set to *p* < 0.002 [29].

Lastly, non-response analyses compared all the children from the initial sample at birth that were not included in the current sample, versus all the children included in the final samples as well as children in the longitudinal sample compared to children in the cross-sectional sample, to assess generalizability of the findings.

## Results

Table 1 summarizes the demographics and behavioral characteristics for the children and their mothers. Additional file 1: Table S1 shows the mean and standard deviation for the brain measures at W1 and W3. Non-response analyses, comparing the initial study sample at birth and the cross-sectional sample W3, showed that children who were not included were more likely to be boys ($\chi$^2^ = 12.93, *df* = 1, *p* = 3.23·10^–4^) and to have a non-Dutch ethnicity ($\chi$^2^ = 76.14, *df* = 2, *p* = 2.20·10^–16^). Mothers of excluded children were on average less educated ($\chi$^2^ = 25.07, *df* = 2, *p* = 3.60·10^–6^) and more likely to have smoked ($\chi$^2^ = 8.75, *df* = 2, *p* = 1.26·10^–2^) and/or consumed alcohol ($\chi$^2^ = 92.97, *df* = 3, *p* = 2.20·10^–16^) during pregnancy. The household income of excluded children tended to be lower ($\chi$^2^ = 27.87, *df* = 2, *p* = 8.88·10^–7^). Non-participants in the longitudinal sample were more likely to be of non-Dutch origin ($\chi$^2^ = 63.34, *df* = 2, *p* = 1.77·10^–14^), come from families with a lower income ($\chi$^2^ = 9.80, *df* = 2, *p* = 7.45·10^–3^), to have mothers with lower education levels ($\chi$^2^ = 9.58, *df* = 2, *p* = 8.33·10^–3^) and to have mothers who consumed more alcohol during pregnancy ($\chi$^2^ = 54.08, *df* = 3, *p* = 7.08·10^–11^). Whereas children with longitudinal data were more likely to be of Dutch ethnicity ($\chi$^2^ = 19.6, *df* = 2, *p* = 5.544·10^–5^) compared to children in the cross-sectional sample, none of the other demographic or participant characteristics were significantly different (see Additional file 1).

**Table 1:** Child and maternal demographic characteristics*

Characteristics	Cross-sectional (*N* = 1950)			Longitudinal (*N* = 304)					
Child	*N*	%/mean	IQR/SD	*N*	%/mean	IQR/SD			
Sex, female (%)	1049	53.8		161	53.0				
Child ethnicity									
Dutch (%)	1229	63.0		233	76.6				
Non western (%)	527	27.0		54	17.8				
Other western (%)	174	8.9		17	5.6				
Missing (%)	20	1.0		0	0				
Handedness									
Right (%)	1747	89.6		271	89.1				
Left (%)	198	10.2		32	10.5				
Missing (%)	5	0.3		1	0.3				
IQ (mean, SD)	1928	97.5	± 12.3	290	97.4	± 11.7			
CBCL attention problems (median, IQR)	1937	2.2	1.0–5.0	192	3.0	1.0–5.5			

	Mean/median	IQR/SD	Range	W1	IQR/SD	Range	W2	IQR/SD	Range
SRS weighted total score (median, IQR)	0.2	0.1–0.3	0.0–2.6	0.2	0.1–0.3	0.0–2.7	0.2	0.1–0.3	0.0–1.9
Age (years) at SRS assessment (mean, SD)	13.5	± 0.4	12.5–16.2	6.2	± 0.5	4.9–8.9	13.5	± 0.4	12.6–16.6
Age (years) at MRI scan (mean, SD)	13.8	± 0.6	12.6–16.4	8.0	± 1.0	6.2–10.7	14.1	± 0.6	13.0–16.6

Maternal	*N*	%	*N*	%					
Education level									
High	1099	56.4	191	62.8					
Medium	623	31.9	104	34.2					
Low	41	2.1	4	1.3					
Missing	187	9.6	5	1.6					
Monthly household income									
High	1495	76.7	244	80.3					
Medium	198	10.2	37	12.2					
Low	104	5.3	10	3.3					
Missing	153	7.8	13	4.3					
Smoking during pregnancy									
Never	1327	67.7	222	73.0					
Until pregnancy was known	147	7.5	17	5.6					
Continued during pregnancy	262	13.3	51	16.8					
Missing	153	11.5	14	4.6					
Alcohol use during pregnancy									
Never	599	30.7	86	28.3					
Until pregnancy was known	243	12.5	38	12.5					
Continued occasionally	640	32.8	115	37.8					
Continued frequently (at least 1 glass	188	9.6	46	15.1					
a week for at least 2 trimesters)									
Missing	280	14.4	19	6.2					

*SRS, Social Responsiveness Scale; CBCL, Child Behavioral Checklist

### Cross-sectional analyses

#### Global morphological measures

The results of the associations between the global morphological brain measures and autistic traits are presented in Table 2. In model 2, autistic traits showed significant negative correlations with cerebellum cortex volume (*ß* = − 0.06, S.E. = 0.02, *p* value = 0.004), mean gyrification (*ß* = − 0.09, S.E. = 0.02, *p* value = 0.005) and mean surface area (*ß* = − 0.09, S.E. = 0.02, *p* value = 0.002). The associations with cerebellum white matter volume (*ß* = − 0.05, S.E. = 0.03, *p* value = 0.019) and mean cortical thickness (*ß* = 0.03, S.E. = 0.02, *p* value = 0.018) did not survive adjustment for multiple comparisons. No significant interactions by sex were observed in these associations (Additional file 1: Table S1).

**Table 2:** Association between global morphological brain measures and autistic traits^a^

Brain measures	Model 1				Model 2			
	*ß*	SD	*t*-statistic	*p* value	*ß*	SD	*t*-statistic	*p* value
Total brain volume	− 0.06	0.88	− 3.16	0.002*	− 0.03	0.88	− 1.69	0.089
Cortical gray matter volume	− 0.06	0.88	− 3.17	0.001*	− 0.03	0.88	− 1.46	0.144
Subcortical gray matter volume	− 0.06	0.88	− 2.79	0.005*	− 0.04	0.88	− 1.77	0.075
Cerebral white matter volume	− 0.04	0.88	− 2.09	0.036	− 0.02	0.88	− 1.12	0.256
CSF volume	− 0.01	0.88	− 0.61	0.540	0.01	0.88	0.54	0.592
Cerebellum cortex volume	− 0.08	0.88	− 4.15	3.39·10^–5^*	− 0.06	0.88	− 2.84	0.004*
Cerebellum white matter volume	− 0.06	0.88	− 2.92	0.003*	− 0.05	0.88	− 2.34	0.019
Mean amygdala volume (L + R)	− 0.05	0.88	− 2.39	0.017	− 0.02	0.88	− 0.96	0.339
Mean gyrification	− 0.09	0.88	− 4.24	2.25·10^–5^*	− 0.06	0.88	− 2.81	0.005*
Mean surface area	− 0.09	0.88	− 4.73	2.44·10^–6^*	− 0.06	0.88	− 3.12	0.002*
Mean cortical thickness	0.03	0.88	1.47	0.142	0.05	0.88	2.38	0.018

^a^Model 1 is adjusted for sex, age at SRS and the difference in age between SRS and MRI; Model 2 is additionally adjusted for child ethnicity, maternal education, maternal smoking and drinking during pregnancy. The effects are standardized

*Significant after Bonferroni correction (*p* < 0.005)

After additionally adjusting for IQ and attention problems in a sensitivity analysis, these associations did not remain statistically significant (Additional file 1: Table S3). In the second sensitivity analysis, children with the highest level of autistic traits or a confirmed ASD diagnosis (*N* = 36) were excluded. We found significant negative associations between autistic traits and mean gyrification (*ß* = − 0.07, S.E. = 0.02, *p* value = 0.0004) and mean surface area (*ß* = − 0.07, S.E. = 0.02, *p* value = 0.0002) (Additional file 1: Table S3).

#### Surface-based measures

**Fig. 1:**
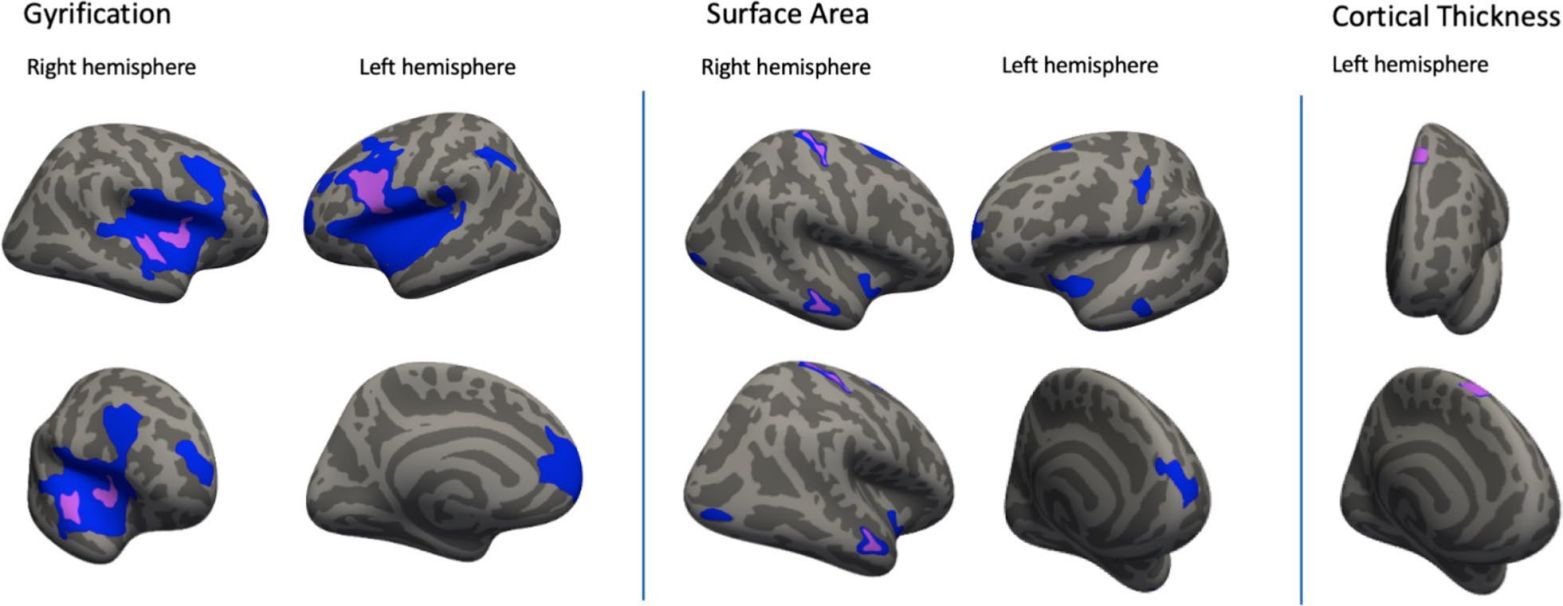
Association between autistic traits and surface-based MRI brain measures in 13- to 16-Year-Old Children^a^. ^a^Regions from Table 3 are depicted in blue for Model 1 and in purple for Model 2. Model 2 was overlaid on model 1. Model 1 is adjusted for sex, handedness, age at SRS and the difference in age between SRS and MRI. Model 2 is additionally adjusted for child ethnicity, maternal education and maternal smoking and drinking during pregnancy

Table 3 and Fig. 1 show the full results of the vertex-wise analyses for the association between autistic traits and surface-based measures of gyrification, surface area and cortical thickness. We found negative associations between autistic traits and gyrification for model 2 in the pars opercularis in the left hemisphere (cluster size = 1105.75 mm^2^, cluster-wise *ß* = − .027); and in the insula (420.30 mm^2^, *ß* = − 0.030) and superior temporal cortex (409.96 mm^2^, *ß* = − 0.031) in the right hemisphere (Fig. 1, Table 3). Two regions showed significant negative associations between autistic traits and surface area for model 2. These include regions in the middle temporal lobe (185.50 mm^2^, *ß* = − 0.012) and the postcentral cortex (185.42 mm^2^, *ß* = − 0.004) of the right hemisphere (Fig. 1, Table 3). Lastly, we found a positive association between autistic traits and cortical thickness for model 2 in a region involving the superior frontal cortex (220.52 mm^2^, *ß* = 0.028) (Fig. 1, Table 3). No significant interactions by sex were observed. After additionally adjusting for IQ and attention problems as a sensitivity analysis, no brain regions remained significant after correcting for multiple testing. After exclusion of the children with the highest level of autistic traits or a confirmed ASD diagnosis (*N* = 36), the significant regions from this analysis showed considerable overlap (mainly in the temporal and frontal lobe) with the regions from the main analysis (Additional file 1: Table S4, Additional file 1: Fig. S3). After additionally correcting for total brain volume, the significant regions cover smaller areas but also show considerable overlap with the regions from the main analysis (Additional file 1: Table S5, Additional file 1: Fig. S4). After additionally correcting the analyses of gyrification, cortical thickness and surface area for mean gyrification, mean cortical thickness, surface area and mean surface area, respectively, there are no significant regions for model 2 (Additional file 1: Table S6, Additional file 1: Fig. S5).

**Table 3:** Vertex-wise results for surface-based brain measures for the left and right hemisphere (LH and RH)^a^

Model	Anatomical region	Area size (mm^2^)	MNI			*N* vertices	Cluster-wise *ß*-value	Cluster-wise *p* value
			*x*	*y*	*z*			
Gyrification LH (*N* = 1927)								
1	Pars opercularis	10,089.32	− 50.3	9.2	5.0	146,256	− 0.031	0.0001
	Superior frontal	3204.78	− 12.5	53.1	3.1	4925	− 0.012	0.0001
	Inferior parietal	1029.14	− 38.2	− 50.8	33.6	2567	− 0.016	0.0001
2	Pars opercularis	1105.75	− 39.8	9.9	22.5	2320	− 0.027	0.0001
Gyrification RH (*N* = 1927)								
1	Superior temporal	7994.05	60.7	− 2.3	− 4.8	18,232	− 0.035	0.0002
	Rostral middle frontal	590.66	23.8	57.5	6.6	804	− 0.012	0.0027
2	Insula	420.30	34.7	5.5	3.6	1040	− 0.030	0.0105
	Superior temporal	409.96	62.2	− 5.8	− 2.9	893	− 0.031	0.0113
Surface area LH (*N* = 1950)								
1	Superior frontal	423.91	− 11.7	64.3	11.3	632	− 0.019	0.0002
	Insula	391.15	− 38.5	− 6.3	− 16.4	1093	− 0.007	0.0002
	Precentral	318.22	− 35.7	− 21.3	36.6	817	− 0.005	0.0003
	Inferior temporal	270.96	− 46.2	− 8.0	− 40.1	446	− 0.022	0.0009
	Middle temporal	230.11	− 57.8	− 17.9	− 17.1	371	− 0.021	0.0027
	Superior frontal	151.19	− 22.2	20.1	54.9	201	− 0.028	0.0228
Surface area RH (*N* = 1950)								
1	Postcentral	506.62	16.9	− 32.9	62.0	1271	− 0.006	0.0001
	Middle temporal	476.04	58.5	− 15.4	− 17.9	767	− 0.015	0.0001
	Superior frontal	334.18	18.5	5.0	62.2	646	− 0.021	0.0001
	Insula	246.06	35.7	− 5.6	− 6.2	596	− 0.008	0.0012
	Lateral occipital	202.13	32.8	− 89.7	− 4.7	245	− 0.019	0.0047
2	Middle temporal	185.50	57.8	− 16.1	− 17.7	289	− 0.012	0.0085
	Postcentral	185.42	16.4	− 33.0	61.4	511	− 0.004	0.0085
Cortical thickness LH (*N* = 1950)								
1	Superior frontal	239.46	− 10.1	24.7	56.7	427	0.029	0.003
2	Superior frontal	220.52	− 10.1	24.7	56.7	397	0.028	0.005

^a^Model 1 is adjusted for sex, handedness, age at SRS and the difference in age between SRS and MRI; Model 2 is additionally adjusted for child ethnicity, maternal education, maternal smoking and drinking during pregnancy. The effects are standardized

### Longitudinal analyses

Table 4 shows the results of the CLPM for autistic traits and MRI findings including global and surface-based measures. The names of the coefficients in this table correspond to the names used in Eq. 1. None of the cross-lagged or cross-sectional pathways showed a significant association between the SRS scores and the brain measures. The autoregressive coefficients showed that autistic traits measured at W1 are positively associated (*ß*≈0.53) with those measured at W3. Furthermore, brain measures at W1 were positively associated with those measured at W3. The fit measures of the models presented in Table 4 are provided in the supplement (Additional file 1: Table S7). After additionally adjusting the global measures for total brain volume and the ROI’s of gyrification, cortical thickness and surface area for mean gyrification, mean cortical thickness and mean surface area, respectively, similarly none of the cross-lagged or cross-sectional pathways showed a significant association (Additional file 1: Table S8). Additional file 1: Table S9 (model 1) and Additional file 1: Table S10 (model 2) shows the results of the analyses including all ROIs from the Desikan-Killiany atlas. None of the cross-lagged pathways are significant. For model 2, only the supra marginal region shows a significant cross-sectional pathway with autistic traits (*ß* = − 0.187, *p* value = 0.001).

**Table 4:** Results Cross-Lagged Panel Model for Global and Surface-based MRI Brain Measures and Autistic Traits^a^

Brain measures	Model	SRS → MRI		MRI → SRS		Cross-Sectional		Autoregressive			
		*ß*_CL-1_	*p* value	*ß*_CL-2_	*p* value	*ß*_CL-baseline_	*p* value	*ß*_AR-SRS_	*p* value	*ß*_AR-MRI_	*p* value
Global (*N* = 304)											
Total brain volume	1	0.010	0.722	− 0.045	0.371	− 0.143	0.007	0.523	6.134·10^–26^*	0.867	6.187·10^–192^*
	2	0.010	0.742	− 0.045	0.374	− 0.098	0.060	0.523	1.087·10^–25^*	0.867	1.391·10^–189^*
Cortical gray matter volume	1	0.015	0.696	− 0.051	0.306	− 0.145	0.008	0.522	9.307·10^–26^*	0.759	3.548·10^–87^*
	2	0.015	0.697	− 0.051	0.310	− 0.099	0.054	0.522	1.675·10^–25^*	0.759	4.011·10^–86^*
Subcortical gray matter volume	1	− 0.047	0.059	− 0.029	0.565	− 0.102	0.057	0.526	3.292·10^–26^*	0.904	5.526·10^–293^*
	2	− 0.047	0.060	− 0.029	0.567	− 0.054	0.278	0.526	5.696·10^–26^*	0.904	1.351·10^–289^*
Cerebral white matter volume	1	− 0.009	0.692	− 0.013	0.796	− 0.133	0.010	0.526	3.587·10^–26^*	0.922	2.437·10^–292^*
	2	− 0.009	0.694	− 0.013	0.797	− 0.092	0.060	0.526	6.286·10^–26^*	0.922	1.838·10^–291^*
Cerebrospinal fluid	1	0.049	0.202	− 0.081	0.101	− 0.064	0.253	0.523	3.781·10^–26^*	0.736	3.136·10^–82^*
	2	0.042	0.442	− 0.048	0.267	− 0.066	0.220	0.523	7.034·10^–26^*	0.736	2.762·10^–81^*
Cerebellum cortex volume	1	0.025	0.394	− 0.039	0.434	− 0.112	0.034	0.525	4.146·10^–26^*	0.867	3.810·10^–195^*
	2	0.025	0.396	− 0.039	0.437	− 0.045	0.357	0.525	7.063·10^–26^*	0.867	6.060·10^–193^*
Cerebellum white matter volume	1	0.019	0.629	− 0.045	0.369	− 0.116	0.036	0.524	5.354·10^–26^*	0.742	9.190·10^–79^*
	2	0.019	0.630	− 0.045	0.371	0.075	0.150	0.524	7.999·10^–26^*	0.742	4.047·10^–78^*
Amygdala volume	1	0.008	0.863	0.009	0.852	− 0.151	0.006	0.528	3.564·10^–26^*	0.586	3.598·10^–35^*
	2	0.008	0.864	0.009	0.853	− 0.090	0.035	0.528	5.966·10^–26^*	0.586	8.120·10^–35^*
Gyrification (*N* = 295)											
Mean gyrification (LH + RH)	1	0.020	0.690	0.009	0.858	− 0.074	0.157	0.527	2.446·10^–26^*	0.863	9.849·10^–199^*
	2	0.020	0.493	0.009	0.858	− 0.045	0.369	0.527	4.315·10^–26^*	0.863	1.506·10^–196^*
Parietal lateral (LH)	1	− 0.001	0.985	− 0.064	0.197	− 0.081	0.138	0.523	5.766·10^–26^*	0.817	3.491·10^–130^*
	2	− 0.001	0.985	− 0.064	0.199	− 0.069	0.191	0.523	9.095·10^–26^*	0.817	4.795·10^–129^*
Occipital (RH)	1	0.035	0.269	0.025	0.616	− 0.100	0.064	0.527	3.000·10^–26^*	0.841	5.854·10^–153^*
	2	0.035	0.271	0.025	0.618	− 0.066	0.200	0.527	5.224·10^–26^*	0.841	2.998·10^–151^*
Surface area (*N* = 304)											
Mean surface area (LH + RH)	1	0.012	0.672	− 0.035	0.471	− 0.147	0.004	0.527	2.243·10^–29^*	0.863	1.289·10^–202^*
	2	0.013	0.643	− 0.033	0.494	− 0.045	0.369	0.535	8.645·10^–29^*	0.876	2.622·10^–199^*
Postcentral medial (LH)	1	− 0.001	0.682	0.480	0.179	0.001	0.912	0.542	2.355·10^–29^*	0.811	4.394·10^–212^*
	2	− 0.001	0.659	0.437	0.217	0.004	0.578	0.534	7.953·10^–29^*	0.880	3.055·10^–223^*
Orbitofrontal (RH)	1	− 0.002	0.679	− 0.041	0.911	− 0.013	0.077	0.546	1.171·10^–29^*	0.781	1.843·10^–133^*
	2	− 0.002	0.607	− 0.076	0.832	− 0.010	0.151	0.537	3.962·10^–29^*	0.779	8.787·10^–132^*
Cortical thickness (*N* = 304)											
Mean cortical thickness (LH + RH)	1	− 0.05	0.928	− 0.039	0.416	− 0.042	0.448	0.543	1.727·10^–29^*	0.928	4.916·10^–19^*
	2	− 0.010	0.493	− 0.038	0.429	− 0.018	0.730	0.534	9.668·10^–29^*	0.436	1.079·10^–17^*
Superior frontal (RH)	1	− 0.006	0.667	− 0.004	0.986	0.023	0.077	0.546	1.762·10^–26^*	0.322	1.238·10^–8^*
	2	− 0.007	0.623	0.052	0.802	0.018	0.161	0.536	1.005·10^–28^*	0.318	2.885·10^–8^*

^a^*ß*_CL-1_ is the cross-lagged path, where SRS scores at W1 predict MRI outcomes at W3; *ß*_CL-2_ is the cross-lagged path between MRI outcomes at W1 and SRS scores at W3; *ß*_CL-baseline_ is the cross-sectional association between MRI outcomes and the SRS within W1; *ß*_AR-SRS_ is the autoregressive coefficient for the SRS score; *ß*_AR-MRI_ is the autoregressive coefficient for the MRI outcomes (see Eq. 1)

^*^Significant after Bonferroni correction (*p* = 0.003)

## Discussion

The main goal of this population-based neuroimaging study was to investigate the bidirectional, longitudinal relationship between autistic traits and brain morphology in children. We found that both autistic traits and structural brain measures are relatively stable across childhood. Contrary to our hypothesis, earlier brain morphology was not associated with later changes in autistic traits. However, in line with our hypotheses, higher levels of autistic traits were cross-sectionally associated with less gyrification, smaller surface area and larger cortical thickness in widespread brain regions. In addition, higher levels of autistic traits were negatively associated with cerebellar cortex volumes, mean gyrification and mean surface area. Most results did not change meaningfully after excluding children with the highest level of autistic traits or confirmed ASD, providing support that this relation extends to subclinical populations. After additionally correcting the vertex-wise analyses for total brain volume, significant regions covered smaller areas but showed considerable overlap with the regions from the main analysis, suggesting the presence of region-specific effects.

Lower gyrification in children with higher levels of autistic traits has also been observed earlier at younger ages within the current sample [4, 5] as well as in other samples [7, 10]. In contrast, studies in the literature also describe higher gyrification in individuals with more autistic symptoms [6, 9], and a recent meta-analysis found no significant differences in gyrification between ASD cases and controls [11]. Given the heterogeneity in symptoms in children with ASD, it has been proposed that individual domains of autistic traits might map differently on the brain. A population-based study of autistic traits in children aged 6-to-18 years, performed an unsupervised clustering analysis that resulted in subgroups with specific behavioral characteristics [9]. They found that the emotional subgroup, characterized by emotional dysregulation and ADHD-symptoms, showed lower gyrification in right precuneus and temporal regions, while the attention and anxiety-depression subgroups showed increased gyrification in occipital and postcentral regions. Future research might benefit from clustering autistic traits based on SRS subscales, making it easier to reproduce and compare findings across studies.

The brain regions of altered gyrification, surface area and cortical thickness that we identified in our cross-sectional analyses at age 13-to-16 years, show substantial overlap with our previous findings in younger age groups within the current sample [4, 5] and other studies in children and adults [7, 12]. In addition, the cerebellum has long been implicated in the neurobiology of ASD [34, 35] and our finding of lower cerebellum cortex volume in children with more autistic traits parallels a recent meta-analysis in adults with ASD that found smaller gray matter volume in the cerebellum [36]. In addition, a recent study of twins with ASD demonstrated that cortical thickness, ventricular volumes, and cerebellar white matter volumes were smaller in children and adolescents with ASD. Interestingly, these findings in the brain were driven by shared environmental factors [37], whereas autistic symptoms have been shown to be more driven by non-shared environment [38]. A recent meta-analysis of social cognition and the cerebellum demonstrated that the cerebellum is involved in mentalizing and understanding people’s intentions based on their movements [39]. In addition to the cerebellum, we also found differences in key brain regions associated with functions that are typically impaired in children with ASD. The insula has been functionally associated with social structure learning [40], which is supported by a meta-analysis of fMRI studies that showed consistent hypoactivation in this region of the brain in individuals with ASD [41]. The pars opercularis, superior temporal cortex, middle temporal cortex and superior frontal cortex, have, among other functions, all been implicated in social cognition, including the perception of mental states and facial expressions of other people [42–45]. This evidence, drawn from the linkage of brain anatomy to brain function, provides support for potential functional abnormalities in ASD in these brain regions.

Interestingly, none of our findings remained significant in our sensitivity analyses in which attention problems and IQ were added as covariates. Both attention problems and cognitive deficits [33] are often comorbid features in children with autistic symptoms. Our findings that the significant brain regions no longer are significant when adding these covariates show that attention problems and IQ differences are not independent of the underlying neurobiology of autistic symptoms, although the neurobiological etiology of these behaviors in children with autistic traits may be different than problems found in other disorders (i.e., ADHD).

In our cross-sectional analyses of global morphological brain measures we found that mean gyrification, mean surface area and cerebellum cortex volume were significantly associated with autistic traits. After excluding children with the highest level of autistic traits or confirmed ASD, mean gyrification and mean surface area were significant, whereas the cerebellar cortex volume was not. In the vertex-wise analyses, the significant regions after exclusion of children with the highest level of autistic traits or ASD show considerable overlap with the regions from the main analysis. However, compared to the regions from the main analysis, regions in our sensitivity analysis were more located in the frontal lobe. Regional differences in gyrification related to autistic symptoms were primarily located in the rostral middle frontal cortex in both the left and right hemispheres and regional differences in surface area were found mostly in the superior frontal cortex in the right hemisphere. For cortical thickness, we found a significant region in the middle temporal cortex in the left hemisphere that was not significant in the main model. This suggests that some brain regions are more specific to characteristic features of autism as opposed to a result of comorbid symptoms.

There are several possible explanations for why we found no significant longitudinal effects. First, and most likely, we observed that both brain morphology and measures of autistic traits are quite stable over time. Thus, the effect we observe cross-sectionally could unfold early in life and remain stable over time. The only longitudinal study investigating ASD and gyrification found an increase in gyrification among boys with ASD in regions in the temporal, frontal and parietal cortex, while gyrification was stable or decreasing in these regions in controls [1]. However, this study was performed in younger children, namely from the age of 3-to-5 years. A second explanation for why we found no cross-lagged relationships could be that our longitudinal sample was smaller than the sample available for our cross-sectional analysis, thus lower statistical power could also contribute to our negative findings. Third, regarding the ROIs, it is possible that regions identified in our independent sample at W2 were not the exact locations where changes take place between W1 and W3, especially if the SRS total scores were driven by different subdomains of autistic traits at different ages. In exploratory, data-driven analyses evaluating all regions of the Desikan-Killiany atlas, while there were no cross-lagged findings following correction for multiple testing, it was interesting that the fusiform gyrus did show uncorrected significance with MRI predicting later autistic symptoms (Additional file 1: Table S9). The fusiform, as well as the enterohinal cortex can be hypothesis-driven targets for future longitudinal studies.

In an earlier longitudinal study evaluating internalizing and externalizing symptoms using a cross-lagged panel model, we found that interestingly, it was not the brain that predicted downstream behavioral changes, but rather behavior that predicted downstream brain changes [15]. This then raises the question whether persisting in specific behavior could result in brain changes, for example as seen with practice effects (e.g., musical practice) [46]. Thus, the absence of cross-lagged associations in our model suggests that this type of brain modeling does not occur in children with autistic traits, but rather the morphological differences remain quite stable between six and fifteen years-of-age. This also suggests that differences seen in cross-sectional studies within this age range likely show stable differences. Our work also highlights the importance of studying individuals with ASD or autistic traits early in life to better gain an understanding of the unfolding of brain/behavior relationships.

The main strength of our study lies in the combination of longitudinal and cross-sectional designs that allows for the examination of developmental trajectories of structural brain measures and autistic traits, as well as the extension and direct comparison of results to our previous findings in younger children. Moreover, the longitudinal data collection of both behavioral and brain morphology measures provided the unique opportunity to assess the bidirectional relationship between brain morphology and autistic traits.

### Limitations

Despite the strengths, our results should also be interpreted in the light of some limitations. Autistic traits were assessed at only two time points, and thus we could not distinguish the within-person versus between-person effects. In addition, assessing autistic traits at more than two timepoints would provide greater reliability of the linear and nonlinear trajectories. Furthermore, two different MRI scanners were used at baseline and follow-up for imaging data acquisition. However, as described elsewhere [15], the scanning process was performed as consistently as possible to minimize the effects of scanner differences. Finally, a data-driven analysis approach using surface-based cross-lagged panel models may yield additional regions. Such an approach is feasible but would require programming to implement.

## Conclusions

In conclusion, our longitudinal and cross-sectional findings, taken together, suggest early changes in brain morphology in children with autistic symptoms that remain quite stable over time. We have demonstrated that during adolescence, higher levels of autistic traits are associated with lower gyrification, smaller surface area, and larger cortical thickness in widespread brain regions and smaller cerebellum cortex volume. Overall, these findings point toward alterations in brain regions involved in social cognition. The majority of our results did not change substantially after excluding children with the highest level of autistic traits or autism spectrum disorder (ASD), providing evidence for the extension of the neurobiology of autistic traits into the general population.

## Supplementary Information

**Additional file 1.** Supplement. Supplemental Methods. A description of the behavioral measurements and imaging quality assessments.** Figure S1**. Derivation of the Study Samples.** Figure S2**. Distribution of the SRS Scores before and after Square Root Transformation.** Table S1**. Mean and Standard Deviation for the Brain Measures.** Table S2**. Association Between Autistic Traits and Global Brain Measures. Testing Sex Interaction Effects.** Table S3**. Sensitivity Analyses for the Association between Global Brain Morphological Measures and Autistic Traits.** Table S4**. Sensitivity Analysis: Vertex-wise Analysis Results After Exclusion of the Children with the Highest Level of Autistic Traits or a Confirmed ASD Diagnosis (N=36).** Figure S3**. Association between Autistic Traits and Surface-based MRI Brain Measures After Exclusion of Children with the Highest Level of Autistic Traits or a Confirmed ASD Diagnosis (N=36).** Table S5**. Vertex-wise Analysis Results adjusted for Total Brain Volume.** Figure S4**. Association between Autistic Traits and Surface-based MRI Brain Measures adjusting for Total Brain Volume.** Table S6**. Vertex-wise Analysis Results adjusting for Mean Gyrification and Mean Cortical Thickness.** Figure S5**. Association between Autistic Traits and Turface-based MRI Brain Measures adjusted for Mean Gyrification and Mean Cortical Thickness.** Table S7**. Fit Measures oF the CLPM.** Table S8**. Results of the Cross-Lagged Panel Model for Global MRI Brain Measures and Autistic Traits Additionally Corrected for Total Brain Volume and Mean Gyrification/Cortical Thickness/Surface Area.** Table S9**. Results of the Cross-Lagged Panel Model for Brain Measures using brain regions derived from the Desikan-Killiany Atlas and Autistic Traits (model 1).** Table S10**. Results of the Cross-Lagged Panel Model using brain regions derived from the Desikan-Killiany Atlas and Autistic Traits (model 2).

## Data Availability

The datasets are only available through protected access, so requests should be directed to the Generation R Study management team (https://generationr.nl/algemeen/organisatie/management-team/). For ethical and privacy restrictions, individual-level data are not publicly available.

## Abbreviations

ASD: Autism spectrum disorder
MRI: Magnetic resonance imaging
SRS: Social responsiveness scale
CSF: Cerebrospinal fluid
QDECR: Query Design Estimate Contrast R package
CLPM: Cross-lagged panel models
ROI: Region of interest
