# Supplementary material for: The longitudinal bidirectional relationship between autistic traits and brain morphology from childhood to adolescence: a population-based cohort study
Source: Mol Autism. 2022 Jul 5;13:31. doi: 10.1186/s13229-022-00504-7 (PMC9258195; doi:10.1186/s13229-022-00504-7)
Supplement: Supplementary file 1 — Additional file 1. Supplement. Supplemental Methods. A description of the behavioral measurements and imaging quality assessments. Figure S1. Derivation of the Study Samples. Figure S2. Distribution of the SRS Scores before and after Square Root Transformation. Table S1. Mean and Standard Deviation for the Brain Measures. Table S2. Association Between Autistic Traits and Global Brain Measures. Testing Sex Interaction Effects. Table S3. Sensitivity Analyses for the Association between Global Brain Morphological Measures and Autistic Traits. Table S4. Sensitivity Analysis: Vertex-wise Analysis Results After Exclusion of the Children with the Highest Level of Autistic Traits or a Confirmed ASD Diagnosis (N=36). Figure S3. Association between Autistic Traits and Surface-based MRI Brain Measures After Exclusion of Children with the Highest Level of Autistic Traits or a Confirmed ASD Diagnosis (N=36). Table S5. Vertex-wise Analysis Results adjusted for Total Brain Volume. Figure S4. Association between Autistic Traits and Surface-based MRI Brain Measures adjusting for Total Brain Volume. Table S6. Vertex-wise Analysis Results adjusting for Mean Gyrification and Mean Cortical Thickness. Figure S5. Association between Autistic Traits and Turface-based MRI Brain Measures adjusted for Mean Gyrification and Mean Cortical Thickness. Table S7. Fit Measures oF the CLPM. Table S8. Results of the Cross-Lagged Panel Model for Global MRI Brain Measures and Autistic Traits Additionally Corrected for Total Brain Volume and Mean Gyrification/Cortical Thickness/Surface Area. Table S9. Results of the Cross-Lagged Panel Model for Brain Measures using brain regions derived from the Desikan-Killiany Atlas and Autistic Traits (model 1). Table S10. Results of the Cross-Lagged Panel Model using brain regions derived from the Desikan-Killiany Atlas and Autistic Traits (model 2). [file 13229_2022_504_MOESM1_ESM.docx]

**Data supplement**

**Supplemental methods**

**The 18-item short form of the Social Responsiveness Scale**

Mothers of the children within the Generation R Study filled in the Social Responsiveness

Scale (SRS) when the children were approximately 6 years of age. The SRS is a questionnaire

that measures autistic traits and was designed for children between 4 and 18 years of age

(1,2). The SRS reflects parental observation of behavior within the past 6 months at the time

of completion. Individual items are scored from 0 (‘never true’) to 3 (‘almost always true’)

with higher scores reflecting greater levels of autistic traits. Symptom domains from the ASD

section of the Diagnostic and Statistical Manual of Mental Disorders, Fifth Edition (DSM-V)

(3) are covered in the SRS, including disorders in social communication & interactions and

restricted/repetitive patterns of behavior, interests, or activities. We used an abbreviated

18-item version of the SRS that has been shown to correlate highly with the full 65-item

version. The correlation between total scores derived by the SRS short form and the

complete SRS in Missouri Twin Study (4) was 0.93 in monozygotic male twins (n= 98) and

0.94 in dizygotic male twins (n = 134). In a sample of 2719 children from the Interactive Autism Network’s (5) the corresponding correlation was 0.99. The SRS was excluded if over 25% of the questions were missing; otherwise a weighted total score was calculated based on the number of non-missing items.

**Autism Spectrum Disorder Diagnosis**

To identify children diagnosed with ASD, medical records from family practice physicians were obtained for children that scored screen positive for ASD. This was based on a screening procedure with three phases. The first phase included children with an SRS weighted total score above 1.078 for boys and 1.000 for girls, the cutoffs recommended for population-based screening (2).

**Image Acquisition**

For the 6-to-9-year-old wave, high resolution T_1_-weighted sequences were obtained using an inversion recovery fast spoiled gradient recalled (IR-FSPGR) sequence with the following parameters: repetition time (TR) = 10.3 ms, echo time (TE) = 4.2 ms, inversion time (TI) = 350 ms, number of excitations (NEX) =1, flip angle = 16 degrees, readout bandwidth = 20.8 kHz, matrix size = 256 x 256, imaging acceleration factor = 2, and the isotropic resolution = 0.9 mm^3^. For the 13- to 16-year-old wave, high resolution T_1_-weighted scans were obtained using an IR-FSPGR sequence with the following parameters: TR = 8.77 ms, TE = 3.4 ms, TI = 600 ms, NEX=1, flip angle = 10 degrees, readout bandwidth = 25 kHz, matrix size = 220 x 220, imaging acceleration factor = 2, and the isotropic resolution = 1.0 mm^3^.

**Image quality assurance**

For the 6- to 9-year-old wave, processed images were inspected to rate the quality of the segmentation on a six-point Likert-scale that ranged from 5 (excellent), 4 (very good), 3 (good), 2 (fair), 1 (poor), and 0 (unusable). We have found that quality assessment of structural MRI scans is best done using a systematic approach. A systematic approach focusing on four different measures, namely the foliation of the cerebellum, gray/white matter interface, evidence of ringing in the image, and finally, quality of the segmentation of subcortical structures (i.e., caudate and putamen) provides a reliable measure of image quality (6). An excellent quality image is when both anterior and posterior foliation of the cerebellum can be seen without blurring. A good quality image has some blurring of the foliation of the cerebellum, but with a clear gray/white matter interface and little to no ringing in both the anterior and posterior brain regions when examining multiple axial sections. A poor image will have blurring of the gray/white matter interface, ringing, and also blurring of the borders of the subcortical region. Scans rated as unusable or poor were excluded from the analyses (7). For the 13- to 16-year-old wave, processed images were assessed by two independent raters who rated each scan on a three-point Likert scale (good, questionable, or poor). Images consistently rated as “poor” were excluded. If disagreement between the raters was present regarding the usability of the data, a final assessment was provided by a third rater or obtained after discussion between expert raters. A subset of the images was assessed by one rater only and compared to automated quality assessment (QA) ratings from an in-house support vector machine learning algorithm. The automated QA had a high sensitivity (83%) and a moderate specificity (74%). Therefore, when both the rater and the automated QA indicated good quality of a scan, the scan was considered to have good quality and included. When the automated QA indicated a scan was not usable or when a disagreement between the rater and the automated QA arose, the scan was assessed by an additional rater to decide on the usability of the scan.

**Covariates**

During pregnancy, data were collected by questionnaire on maternal smoking (‘never’, ‘until pregnancy was known’ and ‘continued during pregnancy”), maternal alcohol use (never, until pregnancy was known, continued drinking occasionally and continued drinking frequently) and maternal education, which was divided into three categories: ‘low’ (no education or only primary school), ‘medium’ (secondary school or lower vocational training) and ‘high’ (higher vocational training, university). Child national origin was based on the birth country of the parents and categorized into: ‘Dutch’, ‘Other-Western’ (European, American Western, Asian Western, Oceanian, and Indonesian) and ‘Non-Western’ (Moroccan, Turkish, Dutch Antilles, Surinamese, Cape Verdean, African, American Non-Western, and Asian Non-Western). Attention problems of the child were quantified using the Child Behavioral Checklist (CBCL) at W1 and W3 (8,9). Cognitive performance was evaluated at W3, using four subsets (Matrix Reasoning, Coding, Digit Span and Vocabulary) of the Wechsler Intelligence Scale for Children (WISC-V), which were used to derive an estimated full-scale IQ (10).

**Non-response**

A non-response analysis comparing the longitudinal sample with the cross-sectional sample was performed. The null hypothesis was not rejected in relation to differences in educational level of the mother ($\chi$^2^=1.263, df=2, p=0.532), household income ($\chi$^2^=0.885, df=2, p=0.642), maternal smoking during pregnancy ($\chi$^2^=3.056, df=2, p=0.217), maternal drinking during pregnancy ($\chi$^2^=7.343, df=3, p=0.062), and sex of the child ($\chi$^2^=0.007, df=1, p=0.932). Children who had longitudinal data were more likely to be of Dutch ethnicity ($\chi$^2^=19.6, df=2, p=5.544×10^-5^).

**Tables and Figures**

**FIGURE S1. Derivation of the Study Samples**


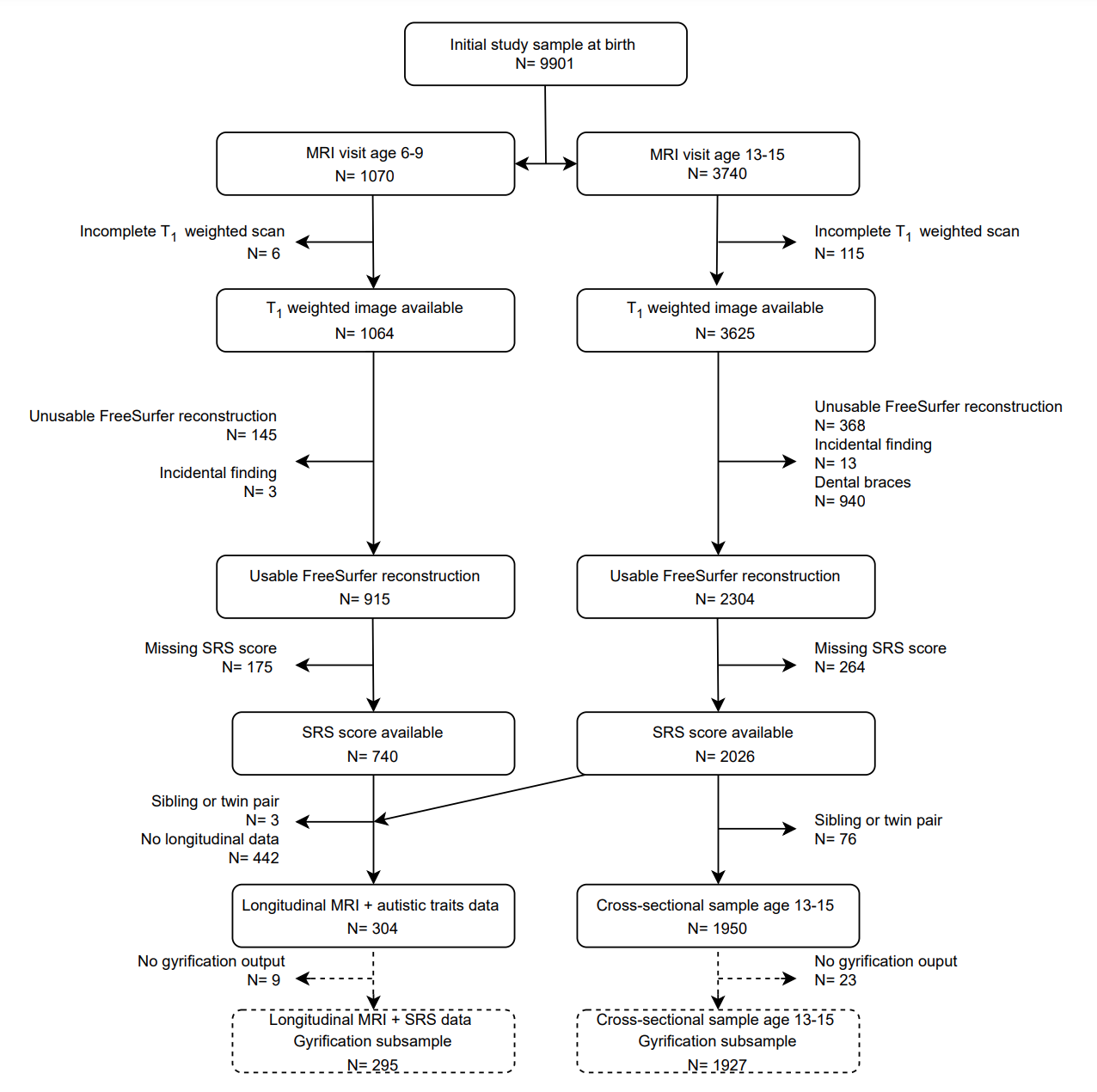


**FIGURE S2. Distribution of the SRS Scores before and after Square Root Transformation**

*
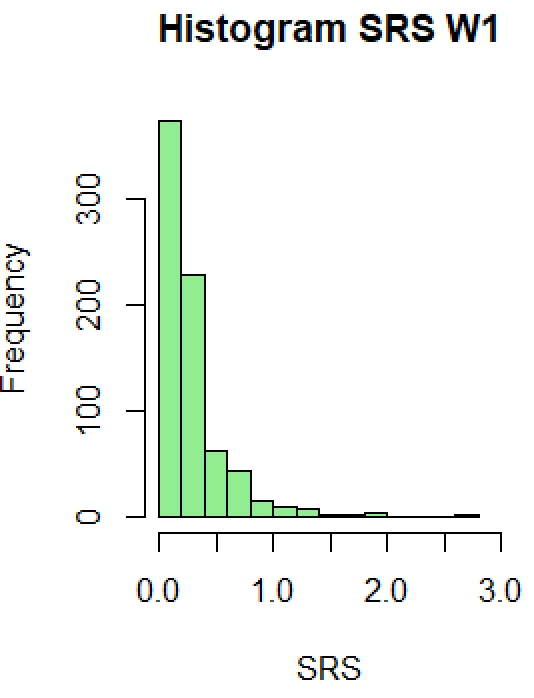

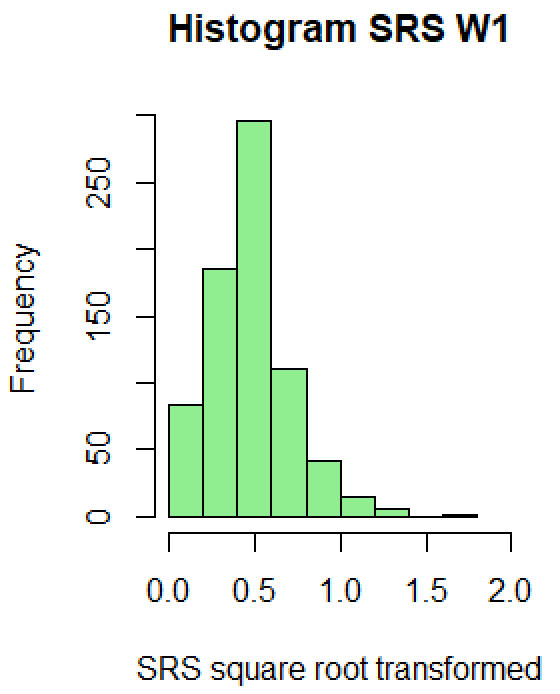
*


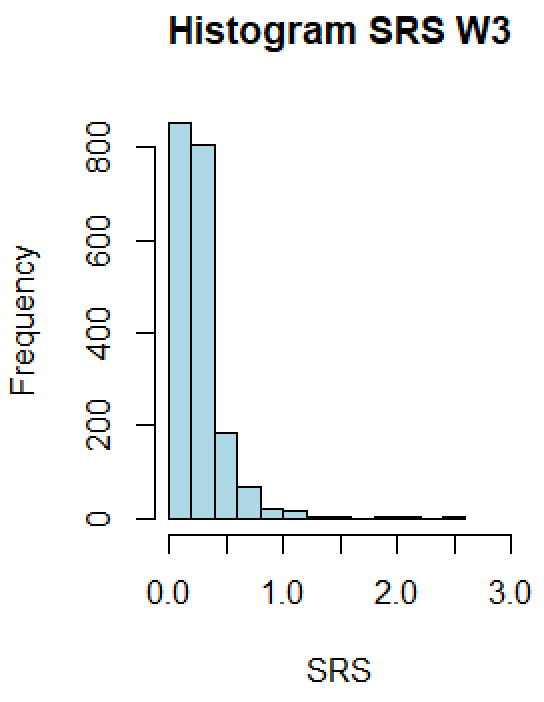

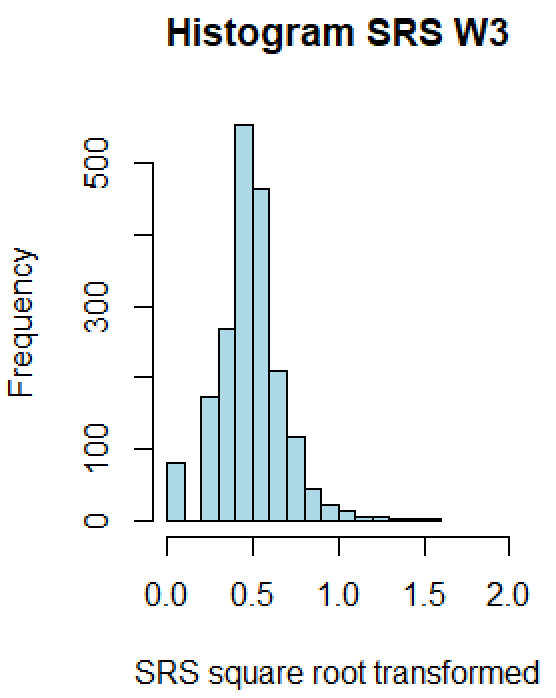


**TABLE S1. Mean and Standard Deviation for the Brain Measures**

| **Brain Measures** |  | |  |  |
| --- | --- | --- | --- | --- |
|  | **Mean W1** | **SD W1** | **Mean W3** | **SD W3** |
| Total Brain Volume (mm^3^) | 1151200 | 121901.4 | 1245481 | 114697.9 |
| Total Gray Matter Volume (mm^3^) | 554678 | 65001.56 | 568775.7 | 53836.66 |
| Subcortical Gray Matter Volume (mm^3^) | 61794.49 | 4905.76 | 61617.18 | 4770.15 |
| Cerebral White Matter Volume (mm^3^) | 382328.3 | 48130.41 | 450876.7 | 51457.02 |
| CSF Volume (mm^3^) | 989.20 | 243.14 | 932.11 | 187.54 |
| Cerebellum Cortex Volume (mm^3^) | 109973.3 | 11085.7 | 122056.8 | 11591.28 |
| Cerebellum White Matter Volume (mm^3^) | 28558.17 | 3549.25 | 26769.14 | 2666.87 |
| Mean Amygdala Volume (L+R) (mm^3^) | 1594.50 | 235.43 | 1804.98 | 195.66 |
| Mean Gyrification (index) | 3.23 | 0.12 | 3.14 | 0.12 |
| Mean Surface Area (mm^2^) | 85336.1 | 9500.75 | 93707.51 | 8822.58 |
| Mean Cortical Thickness (mm) | 2.89 | 0.11 | 2.62 | 0.08 |

**TABLE S2. Association Between Autistic Traits and Global Brain Measures. Testing Sex Interaction Effects**

| **Brain Measures** | **P-value interaction term** | |
| --- | --- | --- |
|  | **Model 1** | **Model 2** |
| Total Brain Volume | 0.03 | 0.12 |
| Total Gray Matter Volume | 0.12 | 0.38 |
| Subcortical Gray Matter Volume | 0.16 | 0.35 |
| Cerebral White Matter Volume | 0.01 | 0.04 |
| CSF Volume | 0.93 | 0.90 |
| Cerebellum Cortex Volume | 0.05 | 0.18 |
| Cerebellum White Matter Volume | 0.35 | 0.53 |
| Mean Amygdala Volume (L+R) | 0.12 | 0.26 |
| Mean Gyrification | 0.81 | 0.51 |
| Mean Surface Area | 0.53 | 0.29 |
| Mean Cortical Thickness | 0.91 | 0.80 |

*Model 1 is adjusted for sex, age at SRS and the difference in age between SRS and MRI; Model 2 is additionally adjusted for child ethnicity, maternal education, maternal smoking and drinking during pregnancy. The effects are standardized, and the Bonferroni corrected p-value is 0.005.

| **TABLE S3. Sensitivity Analyses for the Association between Global Morphological Brain Measures and Autistic Traits^a^** | | | | | | | | |
| --- | --- | --- | --- | --- | --- | --- | --- | --- |
| **Brain Measures** | **Sensitivity analysis 1^a^** | | | | **Sensitivity analysis 2^b^** | | | |
|  | **ß** | **SE** | **t-statistic** | **p-value** | **ß** | **SE** | **t-statistic** | **p-value** |
| Total Brain Volume | 0.002 | 0.02 | 0.12 | 0.258 | -0.05 | 0.02 | -2.62 | 0.009 |
| Total Gray Matter Volume | 0.006 | 0.02 | 0.30 | 0.762 | -0.05 | 0.02 | -2.44 | 0.015 |
| Subcortical Gray Matter Volume | -0.008 | 0.02 | -0.35 | 0.725 | -0.04 | 0.02 | -2.07 | 0.039 |
| Cerebral White Matter Volume | 0.009 | 0.02 | 0.41 | 0.683 | -0.04 | 0.02 | -2.22 | 0.027 |
| CSF Volume | 0.031 | 0.02 | 1.26 | 0.207 | 0.008 | 0.02 | 0.34 | 0.732 |
| Cerebellum Cortex Volume | -0.036 | 0.02 | -1.69 | 0.091 | -0.05 | 0.02 | -2.29 | 0.021 |
| Cerebellum White Matter Volume | -0.019 | 0.02 | -0.85 | 0.397 | -0.05 | 0.02 | -2.31 | 0.021 |
| Amygdala Volume (mean L+R) | -0.010 | 0.02 | -0.44 | 0.662 | -0.05 | 0.02 | -2.47 | 0.013 |
| Mean Gyrification | -0.03 | 0.02 | -1.56 | 0.120 | -0.07 | 0.02 | -3.51 | 0.0004* |
| Mean Surface Area | -0.02 | 0.02 | -1.16 | 0.257 | -0.07 | 0.02 | -3.70 | 0.0002* |
| Mean Cortical Thickness | 0.05 | 0.02 | 2.02 | 0.043 | 0.05 | 0.02 | 2.38 | 0.017 |

^a^ This sensitivity analysis was additionally adjusted for IQ and attention problems (in addition to: sex, age at SRS, the difference in age between SRS and MRI, child ethnicity, maternal education, maternal smoking and drinking during pregnancy). The effects are standardized.

**^b^** In this sensitivity analysis, children with the highest levels of autistic traits (weighted total SRS score above 1.078 for boys and 1.000 for girls) or a confirmed ASD diagnosis were excluded.

* Significant after Bonferroni correction (p=0.005)

**TABLE S4. Sensitivity Analysis: Vertex-wise Analysis Results After Exclusion of the Children with the Highest Level of Autistic Traits or a Confirmed ASD Diagnosis (N=36)**

| **Model** | **Anatomical Region** | **Area Size (mm2)** | **MNI** | | | **N vertices** | | **Cluster-wise**  **ß-value** | **Cluster-wise**  **p-value** |
| --- | --- | --- | --- | --- | --- | --- | --- | --- | --- |
|  |  |  | *x* | *y* | *z* |  |  |  |  |
| **Gyrification LH** | | | | | | | | | |
| *2* | Pars opercularis | 4137.48 | -50.2 | 10.3 | 5.2 | | 9435 | -0.032 | 0.0001 |
|  | Superior frontal | 1347.38 | -9.9 | 48.3 | 9.1 | | 2138 | -0.010 | 0.0001 |
|  | Rostral middle frontal | 949.16 | -28.3 | 33.7 | 23.5 | | 1442 | -0.016 | 0.0001 |
|  | Precentral | 412.83 | -21.6 | -12.3 | 55.2 | | 907 | -0.020 | 0.0124 |
| **Gyrification RH** | | | | | | | | | |
| *2* | Pars opercularis | 3527.00 | 54.8 | 20.9 | 15.0 | | 8018 | -0.034 | 0.0001 |
|  | Lateral orbitofrontal | 1138.74 | 20.3 | 32.9 | 32.9 | | 2169 | -0.013 | 0.0001 |
|  | Rostral middle frontal | 776.43 | 45.4 | 22.7 | 22.7 | | 1525 | -0.024 | 0.0002 |
|  | Rostral middle frontal | 620.92 | 24.2 | 56.3 | 56.3 | | 933 | -0.012 | 0.0015 |
| **Surface area LH** | | | | | | | | | |
| *2* | Inferior temporal | 413.71 | -45.9 | -7.4 | -39.9 | | 664 | -0.025 | 0.0001 |
|  | Rostral middle frontal | 162.76 | -21.6 | 59.9 | 3.4 | | 212 | -0.021 | 0.0162 |
| **Surface area RH** | | | | | | | | | |
| *2* | Middle temporal | 506.62 | 16.9 | -32.9 | 62.0 | | 1271 | -0.016 | 0.0001 |
|  | Superior frontal | 476.04 | 58.5 | -15.4 | -17.9 | | 767 | -0.017 | 0.0001 |
| **Cortical thickness LH** | | | | | | | | | |
| *2* | Middle temporal | 220.09 | -55.6 | -11.0 | -21.9 | | 350 | 0.029 | 0.0052 |
|  | Superior frontal | 171.88 | -9.2 | 23.8 | 58.2 | | 325 | 0.019 | 0.0207 |

**Figure S3. Association between Autistic Traits and Surface-based MRI Brain Measures After Exclusion of the Children with the Highest Level of Autistic Traits or a Confirmed ASD Diagnosis (N=36)^a^**

**
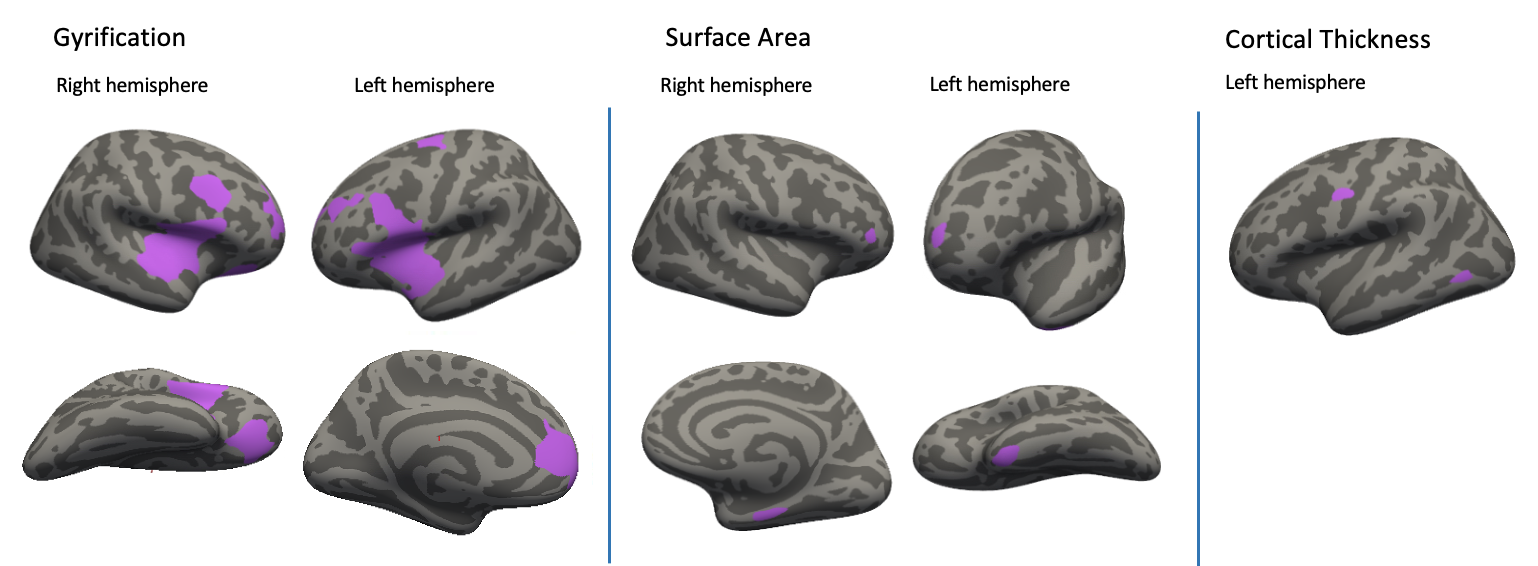
**

^a^ Regions from table S2 are depicted in purple (Model 2). Model 2 is adjusted for sex, handedness, age at SRS and the difference in age between SRS and MRI, child ethnicity, maternal education and maternal smoking and drinking during pregnancy.

**TABLE S5. Vertex-wise Analysis Results with Total Brain Volume Correction**

| **Model** | **Anatomical Region** | **Area Size (mm2)** | **MNI** | | | **N vertices** | | **Cluster-wise**  **ß-value** | **Cluster-wise**  **p-value** |
| --- | --- | --- | --- | --- | --- | --- | --- | --- | --- |
|  |  |  | *x* | *y* | *z* |  |  |  |  |
| **Gyrification LH** | | | | | | | | | |
| *1* | Caudal middle frontal | 1864.50 | -34.9 | 15.1 | 29.2 | | 3981 | -0.025 | 0.0001 |
| *2* | Pars opercularis | 599.33 | -39.2 | 9.8 | 22.6 | | 1281 | -0.026 | 0.0001 |
| **Gyrification RH** | | | | | | | | | |
| *1* | Superior temporal | 2906.70 | 60.4 | -1.9 | -5.3 | | 6791 | -0.029 | 0.0001 |
|  | Caudal middle frontal | 494.40 | 39.3 | 22.5 | 36.2 | | 937 | -0.021 | 0.0054 |
| **Surface area LH** | | | | | | | | | |
| *1* | Inferior temporal | 258.23 | -45.9 | -7.4 | -39.9 | | 429 | -0.021 | 0.0001 |
| *2* | Inferior temporal | 204.46 | -45.8 | -7.4 | -39.8 | | 343 | -0.021 | 0.0002 |
| **Surface area RH** | | | | | | | | | |
| *1* | Middle temporal | 235.72 | 64.4 | -19.5 | -14.5 | | 377 | -0.013 | 0.0001 |
|  | Middle temporal | 181.05 | 54.4 | -5.3 | -29.1 | | 272 | -0.017 | 0.0001 |
| *2* | Middle temporal | 229.94 | 63.9 | -19.3 | -14.4 | | 385 | -0.012 | 0.0009 |
| **Cortical thickness LH** | | | | | | | | | |
| *1* | Superior frontal | 232.51 | -9.6 | 25.0 | 57.0 | | 416 | 0.028 | 0.0038 |
| *2* | Superior frontal | 215.82 | -10.1 | 24.7 | 56.7 | | 390 | 0.028 | 0.0058 |

**Figure S4. Association between Autistic Traits and Surface-based MRI Brain Measures with Total Brain Volume Correction**

**
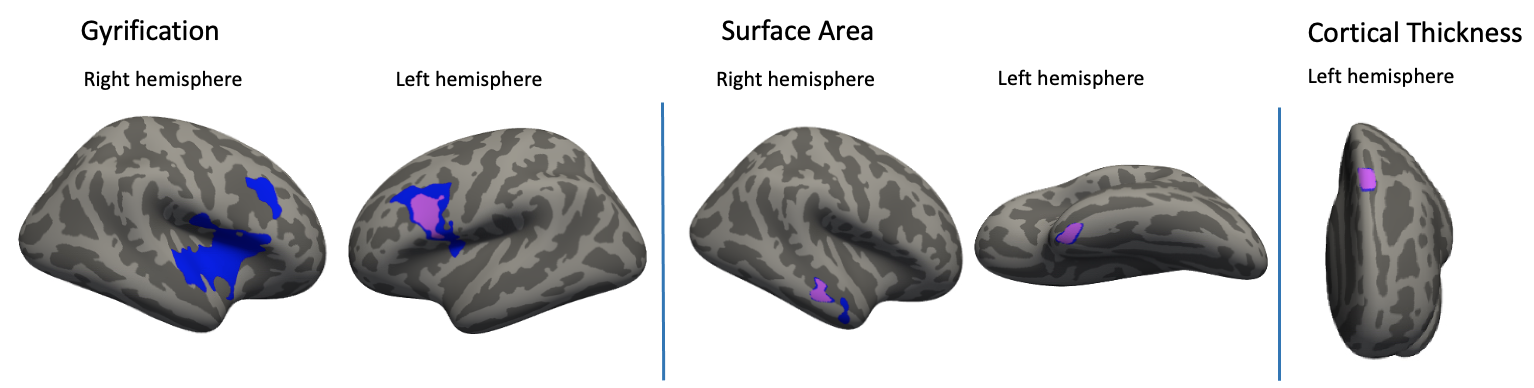
**

^a^ Regions from table S4 are depicted in blue (Model 1) and purple (Model 2). Model 2 was overlaid on model 1. Model 1 is adjusted for sex; age at SRS; the difference in age between SRS and MRI; total brain volume. Model 2 is adjusted for sex; handedness; age at SRS; the difference in age between SRS and MRI; child ethnicity; maternal education; maternal smoking and drinking during pregnancy; total brain volume.

**TABLE S6. Vertex-wise Analysis Results with Mean Gyrification and Mean Cortical Thickness Correction**

| **Model** | **Anatomical Region** | **Area Size (mm2)** | **MNI** | | | **N vertices** | | **Cluster-wise**  **ß-value** | **Cluster-wise**  **p-value** |
| --- | --- | --- | --- | --- | --- | --- | --- | --- | --- |
|  |  |  | *x* | *y* | *z* |  |  |  |  |
| **Gyrification LH** | | | | | | | | | |
| *1* | Lateral occipital | 388.05 | -22.0 | -99.0 | 0.2 | | 454 | 0.015 | 0.0087 |
| **Cortical thickness LH** | | | | | | | | | |
| *1* | Superior frontal | 160.38 | -9.6 | 25.0 | 57.0 | | 416 | 0.026 | 0.018 |
|  |  |  |  |  |  | |  |  |  |

**Figure S5. Association between Autistic Traits and Surface-based MRI Brain Measures with Mean Gyrification and Mean Cortical Thickness correction**


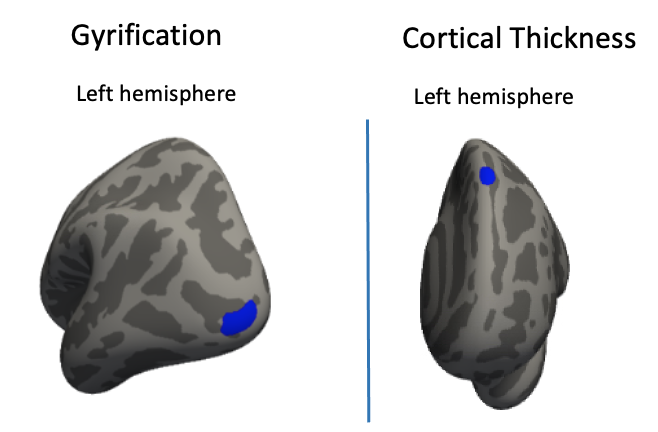


^a^ Regions from table S5 are depicted in blue (Model 1). Model 1 is adjusted for sex; age at SRS; the difference in age between SRS and MRI; mean gyrification (outcome gyrification) or mean cortical thickness (outcome cortical thickness).

**TABLE S7. Fit Measures CLPM**

| **Brain measures** | | |  |  | **Fit Measures^a^** | | | | | | | |
| --- | --- | --- | --- | --- | --- | --- | --- | --- | --- | --- | --- | --- |
|  |  | |  | **Model** | **TLI** | | **CFI** | | **SRMR** | | **RMSEA** | |
| **Global (N= 304)** | | |  |  |  | |  | |  | |  | |
| Total brain volume | |  | | 1 | 0.726 | 0.895 | | 0.042 | | 0.150 | |  |
|  |  |  | | 2 | 0.735 | 0.885 | | 0.032 | | 0.111 | |  |
| Cortical gray matter volume | |  | | 1 | 0.621 | 0.854 | | 0.049 | | 0.149 | |  |
|  |  |  | | 2 | 0.629 | 0.839 | | 0.032 | | 0.112 | |  |
| Subcortical gray matter volume | | | | 1 | 0.844 | 0.940 | | 0.033 | | 0.118 | |  |
|  |  |  | | 2 | 0.836 | 0.928 | | 0.028 | | 0.091 | |  |
| Cerebral white matter volume | | | | 1 | 0.736 | 0.899 | | 0.039 | | 0.168 | |  |
|  |  |  | | 2 | 0.753 | 0.863 | | 0.031 | | 0.099 | |  |
| Cerebrospinal fluid | |  | | 1 | 0.834 | 0.932 | | 0.030 | | 0.093 | |  |
|  |  |  | | 2 | 0.786 | 0.907 | | 0.030 | | 0.078 | |  |
| Cerebellum cortex volume | | | | 1 | 0.721 | 0.893 | | 0.040 | | 0.150 | |  |
|  |  |  | | 2 | 0.711 | 0.874 | | 0.033 | | 0.117 | |  |
| Cerebellum white matter volume | | | | 1 | 0.876 | 0.952 | | 0.037 | | 0.077 | |  |
|  |  |  | | 2 | 0.820 | 0.922 | | 0.031 | | 0.071 | |  |
| Amygdala volume | |  | | 1 | 0.769 | 0.911 | | 0.040 | | 0.091 | |  |
|  |  |  | | 2 | 0.772 | 0.901 | | 0.032 | | 0.069 | |  |
| **Gyrification (N= 295)** | | |  |  |  | |  | |  | |  | |
| Mean gyrification (LH + RH) | | |  | 1 | 0.886 | | 0.956 | | 0.030 | | 0.094 | |
|  |  | |  | 2 | 0.844 | | 0.932 | | 0.028 | | 0.083 | |
| Parietal lateral (LH) | | |  | 1 | 0.880 | | 0.948 | | 0.035 | | 0.080 | |
|  |  | |  | 2 | 0.805 | | 0.910 | | 0.032 | | 0.080 | |
| Occipital (RH) | | |  | 1 | 0.909 | | 0.961 | | 0.029 | | 0.072 | |
|  |  | |  | 2 | 0.879 | | 0.945 | | 0.026 | | 0.065 | |
| **Cortical thickness (N= 304)** | | |  |  |  | |  | |  | |  | |
| Mean cortical thickness (LH + RH) | | |  | 1 | 0.818 | | 0.930 | | 0.037 | | 0.069 | |
|  |  | |  | 2 | 0.763 | | 0.891 | | 0.030 | | 0.059 | |
| Superior frontal (RH) | | |  | 1 | 0.969 | | 0.986 | | 0.028 | | 0.023 | |
|  |  | |  | 2 | 0.837 | | 0.925 | | 0.027 | | 0.044 | |
| **Surface area (N= 304)** | | |  |  |  | |  | |  | |  | |
| Mean surface area (LH + RH) | | |  | 1 | 0.784 | | 0.907 | | 0.038 | | 0.125 | |
|  |  | |  | 2 | 0.769 | | 0.894 | | 0.031 | | 0.100 | |
| Postcentral medial (LH) | | |  | 1 | 0.973 | | 0.988 | | 0.022 | | 0.048 | |
|  |  | |  | 2 | 0.918 | | 0.962 | | 0.024 | | 0.064 | |
| Orbitofrontal (RH) |  | |  | 1 | 0.951 | | 0.979 | | 0.036 | | 0.051 | |
|  |  | |  | 2 | 0.921 | | 0.964 | | 0.029 | | 0.050 | |

^a^  TLI = Tucker-Lewis Index, CFI = comparative fit index, SRMR = standardized root mean square residual, RMSEA = root mean square error of approximation

**TABLE S8. Results Cross-Lagged Panel Model for Global MRI Brain Measures and Autistic Traits Additionally Corrected for Total Brain Volume and Mean Gyrification/Cortical Thickness/Surface Area^a^**

| **Brain measures** | |  |  | **SRS → MRI** |  | **MRI → SRS** |  | **Cross-Sectional** | **p-value** | **Autoregressive** | | | |
| --- | --- | --- | --- | --- | --- | --- | --- | --- | --- | --- | --- | --- | --- |
|  |  |  | **Model** | **ß_CL-1_** | **p-value** | **ß_CL-2_** | **p-value** | **ß_CL-baseline_** |  | **ß_AR-SRS_** | **p-value** | **ß_AR-MRI_** | **p-value** |
| **Global (N= 304) (corrected for total brain volume)** | |  |  |  |  |  |  |  |  |  |  |  |  |
| Cortical gray matter volume | |  | 1 | -0.001 | 0.944 | -0.116 | 0.172 | -0.006 | 0.688 | 0.541 | 4.508×10^-29^* | 0.107 | 0.001* |
|  |  |  | 2 | -0.002 | 0.893 | -0.111 | 0.187 | -0.010 | 0.521 | 0.532 | 1.3403×10^-28^* | 0.105 | 0.001* |
| Subcortical gray matter volume | | | 1 | -0.030 | 0.153 | -0.083 | 0.205 | 0.004 | 0.572 | 0.547 | 5.503×10^-30^* | 0.635 | 7.2627×10^-110^* |
|  |  |  | 2 | -0.029 | 0.170 | -0.061 | 0.347 | -0.054 | 0.552 | 0.539 | 2.0918×10^-29^* | 0.904 | 5.717×10^-106^* |
| Cerebral white matter volume | | | 1 | -0.006 | 0.688 | -0.058 | 0.484 | -0.003 | 0.889 | 0.546 | 8.767×10^-26^* | 0.434 | 1.590×10^-59^* |
|  |  |  | 2 | -0.005 | 0.754 | -0.044 | 0.593 | -0.006 | 0.757 | 0.538 | 2.841×10^-29^* | 0.439 | 1.784×10^-59^* |
| Cerebrospinal fluid | |  | 1 | 0.030 | 0.431 | -0.062 | 0.210 | -0.010 | 0.841 | 0.545 | 7.760×10^-30^* | 0.713 | 1.863×10^-75^* |
|  |  |  | 2 | 0.033 | 0.378 | -0.077 | 0.119 | -0.027 | 0.593 | 0.537 | 2.524×10^-29^* | 0.705 | 1.127×10^-71^* |
| Cerebellum cortex volume | | | 1 | 0.039 | 0.147 | -0.046 | 0.425 | 0.006 | 0.878 | 0.538 | 2.647×10^-29^* | 0.742 | 1.094×10^-114^* |
|  |  |  | 2 | 0.025 | 0.396 | -0.039 | 0.437 | -0.045 | 0.357 | 0.525 | 7.063×10^-26^* | 0.867 | 6.060×10^-114^* |
| Cerebellum white matter volume | | | 1 | 0.024 | 0.490 | -0.004 | 0.945 | -0.005 | 0.915 | 0.547 | 6.301×10^-30^* | 0.628 | 3.103×10^-60^* |
|  |  |  | 2 | 0.024 | 0.507 | -0.020 | 0.696 | -0.003 | 0.950 | 0.539 | 2.069×10^-29^* | 0.629 | 4.796×10^-59^* |
| Amygdala volume | |  | 1 | 0.014 | 0.710 | -0.009 | 0.869 | -0.083 | 0.080 | 0.546 | 1.261×10^-29^* | 0.354 | 2.103×10^-17^* |
|  |  |  | 2 | 0.015 | 0.692 | -0.001 | 0.994 | -0.081 | 0.079 | 0.538 | 3.901×10^-29^* | 0.348 | 9.002×10^-17^* |
| **Gyrification (N= 295) (corrected for mean gyrification)** | |  |  |  |  |  |  |  |  |  |  |  |  |
| Parietal lateral (LH) | |  | 1 | -0.014 | 0.648 | -0.104 | 0.073 | -0.032 | 0.468 | 0.520 | 1.041×10^-25^* | 0.656 | 5.498×10^-76^* |
|  |  |  | 2 | -0.014 | 0.649 | -0.104 | 0.072 | -0.037 | 0.382 | 0.520 | 1.459×10^-25^* | 0.656 | 2.769×10^-76^* |
| Occipital (RH) | |  | 1 | 0.020 | 0.475 | 0.021 | 0.736 | -0.050 | 0.400 | 0.527 | 3.965×10^-26^* | 0.648 | 7.093×10^-80^* |
|  |  |  | 2 | 0.035 | 0.271 | 0.025 | 0.618 | -0.035 | 0.200 | 0.527 | 5.622×10^-26^* | 0.648 | 5.232×10^-79^* |
| **Surface area (N= 304) (corrected for mean surface area)** | |  |  |  |  |  |  |  |  |  |  |  |  |
| Postcentral medial (LH) | |  | 1 | 0.001 | 0.859 | 0.693 | 0.088 | 0.010 | 0.118 | 0.537 | 7.710×10^-29^* | 0.805 | 2.932×10^-307^* |
|  |  |  | 2 | 0.001 | 0.869 | 0.641 | 0.217 | 0.011 | 0.074 | 0.529 | 2.754×10^-28^* | 0.805 | 7.325×10^-301^* |
| Orbitofrontal (RH) |  |  | 1 | -0.001 | 0.754 | -0.102 | 0.814 | 0.001 | 0.984 | 0.546 | 1.007×10^-29^* | 0.686 | 1.833×10^-76^* |
|  |  |  | 2 | -0.002 | 0.690 | -0.144 | 0.738 | 0.001 | 0.995 | 0.538 | 3.302×10^-29^* | 0.685 | 1.887×10^-75^* |
| **Cortical thickness (N= 304) (corrected for mean cortical thickness)** | |  |  |  |  |  |  |  |  |  |  |  |  |
| Superior frontal (RH) | |  | 1 | -0.001 | 0.962 | -0.031 | 0.880 | 0.025 | 0.053 | 0.549 | 9.120×10^-30^* | 0.261 | 4.451×10^-7^* |
|  |  |  | 2 | -0.001 | 0.965 | 0.026 | 0.899 | 0.018 | 0.135 | 0.539 | 4.332×10^-29^* | 0.260 | 6.675×10^-7^* |
|  |  |  |  |  |  |  |  |  |  |  |  |  |  |

^a^ ß_CL-1_ is the cross-lagged path, where SRS scores at W1 predict MRI outcomes at W3; ß_CL-2_ is the cross-lagged path between MRI outcomes at W1 and SRS scores at W3; ß_CL-baseline_ is the cross-sectional association between MRI outcomes and the SRS within W1; ß_AR-SRS_ is the autoregressive coefficient for the SRS score; ß_AR-MRI_ is the autoregressive coefficient for the MRI outcomes (see equation 1).

* Significant after Bonferroni correction (p=0.003)

**TABLE S9. Results Cross-Lagged Panel Model for Brain Measures of Desikan-Killiany Atlas and Autistic Traits^a^ (model 1)**

| **Brain measures** | |  | **SRS → MRI** |  | **MRI → SRS** |  | **Cross-Sectional** | **p-value** | **Autoregressive** | | | |
| --- | --- | --- | --- | --- | --- | --- | --- | --- | --- | --- | --- | --- |
|  |  |  | **ß_CL-1_** | **p-value** | **ß_CL-2_** | **p-value** | **ß_CL-baseline_** |  | **ß_AR-SRS_** | **p-value** | **ß_AR-MRI_** | **p-value** |
| **Global (N= 304)** | |  |  |  |  |  |  |  |  |  |  |  |
| Insula | |  | -0.017 | 0.585 | -0.028 | 0.559 | -0.115 | 0.027 | 0.545 | 9,43×10-27* | 0.836 | 2,00×10-155* |
| Transverse temporal | |  | 0.018 | 0.543 | -0.005 | 0.908 | -0.064 | 0.241 | 0.546 | 6,47×10-27* | 0.846 | 2,91×10-163* |
| Temporal pole | | | -0.098 | 0.073 | -0.031 | 0.515 | 0.515 | 0.096 | 0.543 | 2,25×10-26* | 0.283 | 2.24×10-07* |
| Frontal pole | | | -0.029 | 0.581 | -8.012 | 0.708 | 0.708 | 0.860 | 0.546 | 6,15×10-27* | 0.001 | 1,02×10-06* |
| Supra marginal | |  | -0.027 | 0.477 | -0.047 | 0.322 | -0.187 | 0.001* | 0.540 | 6,49×10-26* | 0.737 | 1,44×10-75* |
| Superior temporal | |  | 0.027 | 0.511 | -0.023 | 0.629 | -0.108 | 0.048 | 0.545 | 1,20×10-26* | 0.701 | 6,24×10-62* |
| Superior parietal | |  | 0.023 | 0.586 | -0.043 | 0.367 | -0.144 | 0.008 | 0.542 | 2,85×10-26* | 0.681 | 6,10×10-55* |
| Superior frontal | |  | 0.027 | 0.456 | -0.048 | 0.314 | -0.101 | 0.055 | 0.544 | 1,17×10-26* | 0.762 | 1,51×10-89* |
| Rostra middle frontal | |  | -0.012 | 0.756 | -0.057 | 0.227 | -0.078 | 0.141 | 0.544 | 8,14×10-27* | 0.694 | 1,17×10-60* |
| Rostral anterior cingulate | |  | 0.000 | 0.994 | -0.054 | 0.256 | -0.102 | 0.055 | 0.544 | 1,14×10-26* | 0.828 | 7,31×10-143* |
| Precuneus | |  | 0.055 | 0.138 | -0.059 | 0.214 | -0.171 | 0.002* | 0.538 | 9,77×10-26* | 0.773 | 1,03×10-92* |
| Precentral | |  | -0.018 | 0.610 | -0.056 | 0.239 | -0.130 | 0.015 | 0.542 | 2,22×10-26* | 0.762 | 4,36×10-91* |
| Posterior cingulate | |  | -0.035 | 0.327 | 0.000 | 0.989 | -0.083 | 0.120 | 0.546 | 6,56×10-27* | 0.776 | 1,84×10-100* |
| Post central | |  | 0.024 | 0.494 | 0.007 | 0.878 | -0.130 | 0.017 | 0.547 | 9,08×10-27* | 0.783 | 3.33×10-105* |
| Pericalcarine | |  | 0.021 | 0.621 | -0.021 | 0.653 | -0.120 | 0.031 | 0.544 | 1,74×10-26* | 0.683 | 2,55×10-56* |
| Pars triangularis | |  | 0.016 | 0.681 | 0.075 | 0.114 | -0.099 | 0.070 | 0.552 | 1,30×10-27* | 0.730 | 2,91×10-74* |
| Pars orbitalis | |  | -0.073 | 0.111 | -0.083 | 0.079 | -0.041 | 0.439 | 0.546 | 3,38×10-27* | 0.600 | 2,66×10-36* |
| Pars opercularis | |  | 0.012 | 0.720 | 0.009 | 0.851 | -0.146 | 0.008 | 0.547 | 1,26×10-26* | 0.811 | 6,79×10-123* |
| Paracentral | |  | -0.088 | 0.050 | 0.023 | 0.618 | -0.066 | 0.228 | 0.547 | 5,36×10-27* | 0.614 | 1,09×10-39* |
| parahippocampal_vol | |  | -0.011 | 0.798 | 0.024 | 0.626 | -0.146 | 0.010 | 0.550 | 1,10×10-26* | 0.682 | 7,37×10-56* |
| middletemporal_vol | |  | 0.040 | 0.358 | -0.071 | 0.138 | -0.125 | 0.020 | 0.540 | 2,83×10-26* | 0.652 | 7,36×10-47* |
| medialorbitofrontal_vol | |  | -0.066 | 0.135 | -0.039 | 0.413 | -0.051 | 0.352 | 0.546 | 6,65×10-27* | 0.633 | 1,50×10-43* |
| lingual_vol | |  | 0.008 | 0.798 | 0.007 | 0.873 | -0.108 | 0.049 | 0.547 | 8,03×10-26* | 0.803 | 9,26×10-118* |
| Lateralorbitofrontal | |  | -0.051 | 0.236 | -0.022 | 0.643 | -0.106 | 0.049 | 0.545 | 1,28×10-26* | 0.648 | 6,59×10-47* |
| Lateraloccipital | |  | 0.046 | 0.193 | -0.003 | 0.945 | -0.062 | 0.244 | 0.546 | 6,42×10-27* | 0.783 | 3,26×10-105* |
| Isthmus cingulate | |  | 0.010 | 0.772 | -0.047 | 0.321 | -0.033 | 0.528 | 0.547 | 4,00×10-27* | 0.784 | 1,67×10-104* |
| Inferior temporal | |  | -0.004 | 0.912 | -0.052 | 0.282 | -0.191 | 0.001* | 0.538 | 2,02×10-25* | 0.689 | 2,14×10-56* |
| Inferior parietal | |  | 0.038 | 0.276 | -0.021 | 0.659 | -0.092 | 0.083 | 0.545 | 9,50×10-27* | 0.781 | 1,47×10-103* |
| Fusiform | |  | 0.022 | 0.584 | -0.107 | 0.025 | -0.115 | 0.034 | 0.539 | 1,76×10-26* | 0.701 | 1.94×10-65* |
| Enterohinal | |  | -0.140 | 0.006 | 0.002 | 0.964 | -0.109 | 0.049 | 0.546 | 1,02×10-26* | 0.423 | 1,29×10-13* |
| Cuneus | |  | 0.057 | 0.175 | -0.003 | 0.939 | -0.116 | 0.030 | 0.546 | 9,00×10-27* | 0.670 | 2,21×10-52* |
| Caudal middle frontal | |  | 0.050 | 0.185 | 0.029 | 0.534 | -0.113 | 0.038 | 0.549 | 5,21×10-27* | 0.757 | 4,06×10-86* |
| Caudal anterior cingulate | |  | -0.010 | 0.655 | -0.024 | 0.625 | -0.167 | 0.003 | 0.543 | 4,73×10-26* | 0.846 | 9,55×10-164* |
| Banks of the superior temporal sulcus volume | |  | 0.043 | 0.307 | -0.056 | 0.243 | -0.105 | 0.054 | 0.543 | 1,39×10-26* | 0.671 | 1,09×10-52* |

^a^ ß_CL-1_ is the cross-lagged path, where SRS scores at W1 predict MRI outcomes at W3; ß_CL-2_ is the cross-lagged path between MRI outcomes at W1 and SRS scores at W3; ß_CL-baseline_ is the cross-sectional association between MRI outcomes and the SRS within W1; ß_AR-SRS_ is the autoregressive coefficient for the SRS score; ß_AR-MRI_ is the autoregressive coefficient for the MRI outcomes (see equation 1).

* Significant after Bonferroni correction (p=0.002)

**TABLE S10. Results Cross-Lagged Panel Model for Brain Measures of Desikan-Killiany Atlas and Autistic Traits^a^ (model 2)**

| **Brain measures** | |  | **SRS → MRI** |  | **MRI → SRS** |  | **Cross-Sectional** | **p-value** | **Autoregressive** | | | |
| --- | --- | --- | --- | --- | --- | --- | --- | --- | --- | --- | --- | --- |
|  |  |  | **ß_CL-1_** | **p-value** | **ß_CL-2_** | **p-value** | **ß_CL-baseline_** |  | **ß_AR-SRS_** | **p-value** | **ß_AR-MRI_** | **p-value** |
| **Global (N= 304)** | |  |  |  |  |  |  |  |  |  |  |  |
| Insula | |  | -0.017 | 0.586 | -0.028 | 0.559 | -0.115 | 0.027 | 0.545 | 9,43×10--27* | 0.836 | 2,01×10-155* |
| Transverse temporal | |  | 0.019 | 0.544 | -0.006 | 0.909 | -0.064 | 0.241 | 0.547 | 6,47×10--27* | 0.847 | 2,92×10-163* |
| Temporal pole | | | -0.098 | 0.073 | -0.031 | 0.516 | -0.092 | 0.096 | 0.544 | 2,25×10--26* | 0.284 | 2,24×10-04* |
| Frontal pole | | | -0.029 | 0.581 | -0.013 | 0.709 | -2.159 | 0.860 | 0.547 | 6,15×10--27* | 0.001 | 1,03×10-06* |
| Supra marginal | |  | -0.028 | 0.478 | -0.048 | 0.322 | -0.187 | 0.001* | 0.541 | 6,49×10--26* | 0.737 | 1,44×10-75* |
| Superior temporal | |  | 0.027 | 0.511 | -0.023 | 0.629 | -0.108 | 0.048 | 0.545 | 1,20×10--26* | 0.701 | 6,24×10-62* |
| Superior parietal | |  | 0.023 | 0.587 | -0.044 | 0.367 | -0.144 | 0.009 | 0.543 | 2,85×10--26* | 0.681 | 6,10×10-55* |
| Superior frontal | |  | 0.028 | 0.456 | -0.048 | 0.314 | -0.101 | 0.055 | 0.544 | 1,18×10--26* | 0.762 | 1,51×10-89* |
| Rostra middle frontal | |  | -0.013 | 0.756 | -0.058 | 0.228 | -0.078 | 0.141 | 0.545 | 8,14×10--27* | 0.694 | 1,17×10-60* |
| Rostral anterior cingulate | |  | 0.000 | 0.994 | -0.055 | 0.256 | -0.102 | 0.056 | 0.544 | 1,14×10--26* | 0.828 | 7,31×10-143* |
| Precuneus | |  | 0.055 | 0.138 | -0.060 | 0.215 | -0.171 | 0.056 | 0.539 | 9,77×10--26* | 0.773 | 1,03×10-92* |
| Precentral | |  | -0.019 | 0.610 | -0.057 | 0.239 | -0.130 | 0.056 | 0.542 | 2,21×10-26* | 0.762 | 4,36×10-91* |
| Posterior cingulate | |  | -0.035 | 0.327 | -0.007 | 0.989 | -0.083 | 0.056 | 0.547 | 6,56×10-27* | 0.777 | 1,84×10-100* |
| Post central | |  | 0.025 | 0.495 | 0.007 | 0.879 | -0.130 | 0.056 | 0.547 | 9,08×10-27* | 0.783 | 3,34×10-102* |
| Pericalcarine | |  | 0.021 | 0.621 | -0.022 | 0.653 | -0.120 | 0.056 | 0.545 | 1,74×10-26* | 0.684 | 2,55×10-56* |
| Pars triangularis | |  | 0.016 | 0.686 | 0.076 | 0.115 | -0.099 | 0.056 | 0.553 | 1,30×10-27* | 0.730 | 2,91×10-74* |
| Pars orbitalis | |  | -0.073 | 0.112 | -0.084 | 0.080 | -0.041 | 0.056 | 0.547 | 3,38×10-27* | 0.600 | 2,66×10-36* |
| Pars opercularis | |  | 0.012 | 0.720 | 0.009 | 0.852 | -0.146 | 0.057 | 0.548 | 1,26×10-26* | 0.811 | 6,79×10-123* |
| Paracentral | |  | -0.088 | 0.050 | 0.024 | 0.619 | -0.066 | 0.057 | 0.548 | 5,36×10-27* | 0.614 | 1,09×10-39* |
| parahippocampal_vol | |  | -0.011 | 0.799 | 0.024 | 0.627 | -0.146 | 0.056 | 0.550 | 1,10×10-26* | 0.683 | 7,37×10-56* |
| middletemporal_vol | |  | 0.040 | 0.359 | -0.071 | 0.138 | -0.125 | 0.057 | 0.541 | 2,83×10-26* | 0.652 | 7,36×10-47* |
| medialorbitofrontal_vol | |  | -0.066 | 0.136 | -0.039 | 0.413 | -0.051 | 0.057 | 0.546 | 6,65×10-27* | 0.633 | 1,50×10-43* |
| lingual_vol | |  | 0.009 | 0.798 | 0.008 | 0.874 | -0.108 | 0.057 | 0.547 | 8,03×10-27* | 0.803 | 9,26×10-118* |
| Lateralorbitofrontal | |  | -0.052 | 0.237 | -0.022 | 0.643 | -0.106 | 0.057 | 0.545 | 1,28×10-26* | 0.648 | 6,59×10-47* |
| Lateraloccipital | |  | 0.046 | 0.193 | -0.003 | 0.945 | -0.062 | 0.057 | 0.547 | 6,42×10-27* | 0.784 | 3,26×10-105* |
| Isthmus cingulate | |  | 0.010 | 0.773 | -0.048 | 0.321 | -0.033 | 0.057 | 0.548 | 4,00×10-27* | 0.784 | 1,67×10-104* |
| Inferior temporal | |  | -0.005 | 0.913 | -0.052 | 0.282 | -0.191 | 0.058 | 0.539 | 2,01×10-25* | 0.689 | 2,14×10-56* |
| Inferior parietal | |  | 0.039 | 0.276 | -0.021 | 0.660 | -0.092 | 0.058 | 0.546 | 9,50×10-27* | 0.781 | 1,47×10-103* |
| Fusiform | |  | 0.022 | 0.585 | -0.107 | 0.025 | -0.115 | 0.058 | 0.540 | 1,76×10-26* | 0.701 | 1,94×10-62* |
| Enterohinal | |  | -0.140 | 0.006 | 0.002 | 0.964 | -0.109 | 0.058 | 0.547 | 1,02×10-26* | 0.423 | 1,29×10-13* |
| Cuneus | |  | 0.058 | 0.176 | -0.004 | 0.939 | -0.116 | 0.058 | 0.547 | 9,00×10-27* | 0.670 | 2,21×10-52* |
| Caudal middle frontal | |  | 0.050 | 0.186 | 0.030 | 0.535 | -0.113 | 0.058 | 0.549 | 5,21×10-27* | 0.757 | 4,06×10-86* |
| Caudal anterior cingulate | |  | -0.014 | 0.656 | -0.024 | 0.626 | -0.167 | 0.058 | 0.544 | 4,73×10-26* | 0.846 | 9,55×10-164* |
| Banks of the superior temporal sulcus volume | |  | 0.044 | 0.308 | -0.056 | 0.243 | -0.105 | 0.058 | 0.543 | 1,39×10-26* | 0.671 | 1,09×10-52* |

^a^ ß_CL-1_ is the cross-lagged path, where SRS scores at W1 predict MRI outcomes at W3; ß_CL-2_ is the cross-lagged path between MRI outcomes at W1 and SRS scores at W3; ß_CL-baseline_ is the cross-sectional association between MRI outcomes and the SRS within W1; ß_AR-SRS_ is the autoregressive coefficient for the SRS score; ß_AR-MRI_ is the autoregressive coefficient for the MRI outcomes (see equation 1).

* Significant after Bonferroni correction (p=0.002)

**References**

1. Constantino JN, Przybeck T, Friesen D, Todd RD. Reciprocal social behavior in children with and without pervasive developmental disorders. Vol. 21, Journal of Developmental and Behavioral Pediatrics. 2000. p. 2–11.

2. Constantino JN GC. Social responsiveness scale (SRS); manual. West Psychol Serv. 2005;

3. Diagnostic and Statistical Manual of Mental Disorders: DSM-5. 5th ed. Am Psychiatr Assoc. 2013.

4. Ronald A, Hoekstra RA. Autism spectrum disorders and autistic traits: A decade of new twin studies. Am J Med Genet Part B Neuropsychiatr Genet. 2011;156(3):255–74.

5. Lyall K, Hosseini M, Ladd-Acosta C, Ning X, Catellier D, Constantino JN, et al. Distributional Properties and Criterion Validity of a Shortened Version of the Social Responsiveness Scale: Results from the ECHO Program and Implications for Social Communication Research. J Autism Dev Disord. 2021;51(7):2241–53.

6. White T, Jansen PR, Muetzel RL, Sudre G, El Marroun H, Tiemeier H, et al. Automated quality assessment of structural magnetic resonance images in children: Comparison with visual inspection and surface-based reconstruction. Hum Brain Mapp. 2018;39(3):1218–31.

7. Blanken LME, Mous SE, Ghassabian A, Muetzel RL, Schoemaker NK, El Marroun H, et al. Cortical morphology in 6- to 10-year old children with autistic traits: a population-based neuroimaging study. Am J Psychiatry. 2015 May;172(5):479–86.

8. Achenbach, T. M., & Rescorla LA. Manual for the ASEBA preschool forms and profiles. 2000;Vol. 30(Burlington, VT: University of Vermont, Research center for children, youth, families).

9. Achenbach, T. M., & Rescorla L. Manual for the ASEBA school-age forms & profiles: An integrated system of multi-informant assessment. 2001;(Burlington, VT:: Aseba.).

10. A. S. Kaufman, S. E. Raiford, D. L. Coalson. Intelligent testing with the WISC-V. John Wiley Sons. 2015;
